# 11β-hydroxysteroid dehydrogenase-1 deficiency alters brain energy metabolism in acute systemic inflammation

**DOI:** 10.1016/j.bbi.2017.11.015

**Published:** 2018-03

**Authors:** Manu Verma, Tiina M.J. Kipari, Zhenguang Zhang, Tak Yung Man, Thorsten Forster, Natalie Z.M. Homer, Jonathan R. Seckl, Megan C. Holmes, Karen E. Chapman

**Affiliations:** a University/BHF Centre for Cardiovascular Science, The Queen’s Medical Research Institute, 47 Little France Crescent, Edinburgh EH16 4TJ, UK; b MRC Centre for Inflammation Research, The Queen’s Medical Research Institute, 47 Little France Crescent, Edinburgh EH16 4TJ, UK; c Division of Infection and Pathway Medicine, University of Edinburgh, The Chancellor’s Building, 49 Little France Crescent, Edinburgh EH16 4SB, UK; d Mass Spectrometry Core, Edinburgh Clinical Research Facility, University of Edinburgh, The Queen’s Medical Research Institute, 47 Little France Crescent, Edinburgh EH16 4TJ, UK

**Keywords:** 3PGA, 3-phosphoglyceraldehyde, 11β-HSD1, 11β-hydroxysteroid dehydrogenase type-1, 11-DHC, 11-dehydrocorticosterone, DHAP, dihydroxyacetone phosphate, HPA, hypothalamicpituitary-adrenal, LPS, lipopolysaccharide, OAA, oxaloacetic acid, TCA, tricarboxylic acid, Glucocorticoid metabolism, Inflammation, Glycolysis, 11β-hydroxysteroid dehydrogenase, Energy metabolism

## Abstract

Chronically elevated glucocorticoid levels impair cognition and are pro-inflammatory in the brain. Deficiency or inhibition of 11β-hydroxysteroid dehydrogenase type-1 (11β-HSD1), which converts inactive into active glucocorticoids, protects against glucocorticoid-associated chronic stress- or age-related cognitive impairment. Here, we hypothesised that 11β-HSD1 deficiency attenuates the brain cytokine response to inflammation. Because inflammation is associated with altered energy metabolism, we also examined the effects of 11β-HSD1 deficiency upon hippocampal energy metabolism.

Inflammation was induced in 11β-HSD1 deficient (*Hsd11b1^Del/Del^*) and C57BL/6 control mice by intraperitoneal injection of lipopolysaccharide (LPS). LPS reduced circulating neutrophil and monocyte numbers and increased plasma corticosterone levels equally in C57BL/6 and *Hsd11b1^Del/Del^* mice, suggesting a similar peripheral inflammatory response. However, the induction of pro-inflammatory cytokine mRNAs in the hippocampus was attenuated in *Hsd11b1^Del/Del^* mice. Principal component analysis of mRNA expression revealed a distinct metabolic response to LPS in hippocampus of *Hsd11b1^Del/Del^* mice. Expression of *Pfkfb3* and *Ldha,* key contributors to the Warburg effect, showed greater induction in *Hsd11b1^Del/Del^* mice. Consistent with increased glycolytic flux, levels of 3-phosphoglyceraldehyde and dihydroxyacetone phosphate were reduced in hippocampus of LPS injected *Hsd11b1^Del/Del^* mice. Expression of *Sdha* and *Sdhb*, encoding subunits of succinate dehydrogenase/complex II that determines mitochondrial reserve respiratory capacity, was induced specifically in hippocampus of LPS injected *Hsd11b1^Del/Del^* mice, together with increased levels of its product, fumarate.

These data suggest 11β-HSD1 deficiency attenuates the hippocampal pro-inflammatory response to LPS, associated with increased capacity for aerobic glycolysis and mitochondrial ATP generation. This may provide better metabolic support and be neuroprotective during systemic inflammation or aging.

## Highlights

• 11β-HSD1-deficiency reduces the hippocampal inflammatory response to LPS.
• This happens despite similar peripheral inflammation.
• 11β-HSD1-deficiency favours a “Warburg” like response to LPS in the hippocampus.
• LPS increases hippocampal fumarate levels in 11β-HSD1-deficient mice.
• Fumarate accumulation can cause pseudo-hypoxia.

## 1 Introduction

Although glucocorticoids exert powerful anti-inflammatory effects, chronically elevated glucocorticoid levels are associated with pro-inflammatory conditions in humans and rodents, including cognitive impairment and hippocampal dysfunction (Lupien et al., 1998, Starkman et al., 2001). Prior stress or elevated glucocorticoid levels, including during aging, potentiate the brain pro-inflammatory cytokine response to bacterial lipopolysaccharide (LPS) (Frank et al., 2010, Frank et al., 2012, Barrientos et al., 2015). Even a modest increase in basal glucocorticoid levels in rodents is associated with greater CNS accumulation of pro-inflammatory markers (Perez-Nievas et al., 2007). In rodents, adrenalectomy combined with fixed low dose glucocorticoid replacement from mid-life can prevent the decline in cognitive function associated with age-related elevation of glucocorticoid levels (Landfield et al., 1981).

11β-hydroxysteroid dehydrogenase type-1 (11β-HSD1) increases intracellular glucocorticoid levels by regenerating active glucocorticoids (predominantly cortisol in humans, corticosterone in rats and mice) from intrinsically inert forms (cortisone and 11-dehydrocorticosterone, respectively) (Chapman et al., 2013). 11β-HSD1 deficient mice maintain normal circulating levels of corticosterone and have increased adrenal gland size, to compensate for the lack of tissue reactivation of active glucocorticoids (Carter et al., 2009). 11β-HSD1 mediates many of the adverse cognitive and metabolic effects of circulating corticosterone or cortisol excess (Chapman et al., 2013, Morgan et al., 2014, Yau et al., 2015). 11β-HSD1-deficiency or inhibition is protective against age and/or stress related cognitive decline in rodents (Sooy et al., 2010, Yau et al., 2007, Yau et al., 2001, Yau et al., 2011, Mohler et al., 2011), associated with reduced intra-hippocampal levels of corticosterone (Yau et al., 2015, Yau et al., 2015). Conversely, transgenic over expression of 11β-HSD1 in the forebrain of mice advances age related cognitive impairment (Holmes et al., 2010). However, the molecular mechanisms underlying the protective effects of 11β-HSD1-deficiency against the adverse centrally mediated effects of glucocorticoids remain unknown.

In the periphery, 11β-HSD1-deficiency alters intermediary metabolism and cellular glucose uptake (Morton et al., 2001, Morton et al., 2004, Wamil et al., 2011). These effects are particularly marked when circulating glucocorticoid levels are elevated (Morgan et al., 2014). Glucocorticoids are well known to affect glucose utilization in the CNS (Doyle et al., 1994, de Leon et al., 1997, Brunetti et al., 1998). Moreover, recent evidence has highlighted the importance of a metabolic switch from oxidative respiration to aerobic glycolysis in stress-related inflammation or the aging brain (Currais, 2015). These findings suggest that 11β-HSD1 may alter brain energy metabolism, especially when the hypothalamic–pituitaryadrenal (HPA) axis is activated. 11β-HSD1 is widely expressed, including in resident macrophages (Gilmour et al., 2006, Zhang et al., 2017) and other immune cells, where its expression is increased following cellular activation (Coutinho et al., 2016) (reviewed in Chapman et al., 2013). In many tissues, 11β-HSD1 is up-regulated at sites of inflammation (Bryndova et al., 2004, Ergang et al., 2011), though it remains unchanged at others (Dover et al., 2007). It is highly induced by IL-1 and TNFα, and by glucocorticoids, but again, not in all cells and tissues (Escher et al., 1997, Tomlinson et al., 2001, Cai et al., 2001, Cooper et al., 2001, Sai et al., 2009, Sai et al., 2008) (reviewed in Chapman et al., 2013). 11β-HSD1 modulates the inflammatory response in a site and context-specific manner, with either beneficial or adverse effects upon the severity and resolution of inflammation (reviewed, Chapman et al., 2013).

Here we have tested the hypothesis that deficiency in 11β-HSD1 is protective against a central pro-inflammatory state induced by peripheral administration of LPS, a potent inflammatory stimulus and activator of the HPA axis (Silverman and Sternberg, 2012). We also predicted that an attenuated pro-inflammatory cytokine response in the brain would be associated with altered brain energy metabolism. We therefore examined expression of key enzymes known to contribute to aerobic glycolysis (or the Warburg effect), enzymes involved in oxidative metabolism and substrate transporters (glucose and lactate). We focussed on the hippocampus because of a considerable body of work implicating it as a key locus for the neuroprotective effects of 11β-HSD1 deficiency/inhibition following stress or during aging (Yau et al., 2015, Yau et al., 2007, Yau et al., 2011, Yau et al., 2015, Holmes et al., 2010, Sarabdjitsingh et al., 2014). Bacterial LPS was used to induce peritonitis and acute systemic inflammation in global 11β-HSD1 deficient mice (*Hsd11b1^Del/Del^*). Sickness behaviour, pro-inflammatory response and the associated alteration in energy metabolising pathways were investigated.

## 2 Materials and methods

### 2.1 Animals

Animal studies were conducted in strict accordance with the UK Home Office Animals (Scientific procedure) Act 1986, amended in 2012 to comply with the European Directive 2010/63/EU. Studies were conducted with prior approval from the University of Edinburgh Animal Welfare and Ethical Review Body. Mice were group housed in a controlled environment (25 °C, 12 h:12 h light dark cycle) and provided with standard chow diet and water *ad libitum*.

Mice homozygous for a null allele of *Hsd11b1* (termed *Del*), here referred to as *Hsd11b1^Del/Del^*, were generated by *Cre-LoxP* mediated deletion of exon 3 of the *Hsd11b1* gene. Briefly, *Hsd11b1^f/f^* mice, homozygous for a “floxed” allele of *Hsd11b1* in which exon 3 is flanked by *LoxP* sites, were generated in a C57BL/6 genetic background by TaconicArtemis, Denmark. *Hsd11b1^f/f^* mice were crossed with mice expressing *Cre* recombinase from the *Hprt* locus (expressed in the germ line) (Tang et al., 2002) to create *Hsd11b1^+/Del^* mice, with germ line disruption of *Hsd11b1*. *Hsd11b1^+/Del^* mice were subsequently backcrossed to C57BL/6 for at least five generations before generating homozygous *Hsd11b1^Del/Del^* mice. Background strain characterisation of *Hsd11b1^Del/Del^* mice against a panel of SNP markers covering all chromosomes confirmed that *Hsd11b1^Del/Del^* mice show 99.93–100% similarity to the C57Bl/6 reference genome (Vandermosten et al., in press). Assays of 11β-HSD enzyme activity confirmed that *Hsd11b1^Del/Del^* mice lack 11β-HSD activity in all tissues tested: the brain, liver, adipose tissue, lung and peritoneal cells (Supplementary Fig. 1).

Male mice, aged 10–20 weeks, were used in experiments. Mice were euthanised by CO_2_ asphyxiation, unless stated otherwise. Brains were removed and hippocampi dissected on wet ice, snap frozen on dry ice (for RNA extraction) or in liquid nitrogen (for metabolite measurement) and stored at −80 °C until analysis. Spleens were dissected and weighed.

### 2.2 Sickness behaviour assay

Burrowing activity was quantified to assess sickness behaviour as described (Deacon, 2006, Deacon, 2012). Burrowing behaviour is dependent upon an intact hippocampus and, to a lesser extent, prefrontal cortex (Deacon et al., 2002, Deacon et al., 2003). Briefly, 150 g of food pellets were placed in a burrowing tube sealed at one end (20 cm in length; 6.8 cm in diameter, with screws at one end at an angle of 90° elevating the open end by 3 cm). The amount of food pellets burrowed overnight (for habituation) or over a 2 h period (baseline or during an experiment) was calculated from the weight of food pellets left in the burrowing tube at the end of the assay. Baseline burrowing activity was measured over 2 periods of 2 h, 48 h apart. Representative burrowing activity in C57BL/6 and *Hsd11b1^Del/Del^* mice is shown in Supplementary Fig. 2, with no difference observed between genotypes.

### 2.3 LPS administration

Baseline sickness behaviour was assessed in C57BL/6 and *Hsd11b1^Del/Del^* mice prior to pseudo-randomisation into experimental groups. C57BL/6 and *Hsd11b1^Del/Del^* mice were injected (intra-peritoneal) with 100 µg/kg LPS (Sigma-Aldrich, Dorset, UK) or vehicle (0.9% saline) between 7:00 h and 09:45 h. Mice were euthanised by decapitation 3 h, 6 h or 9 h later. Trunk blood was collected for the measurement of plasma corticosterone, 11-dehydrocorticosterone and circulating leukocytes. Sickness behaviour was assessed in the 2 h prior to kill. Tissues were collected as described above.

### 2.4 Flow cytometry

Quantification of circulating blood leukocytes was performed by flow cytometry as described (Kipari et al., 2013). Briefly, 30 µl of trunk blood was added to 30 µl of sodium citrate (3.9% w/v). Red blood cells were lysed by addition of 0.5 ml of FACS^TM^ lysis buffer (BD Biosciences, Oxford, UK), according to the manufacturer’s protocol. Fluorescent conjugated antibodies (100 ng each, in a total of 50 µl PBS) were added and incubated on ice for 25 min. The antibodies used were: CD45-PE (30-F11; Biolegend, UK), CD11b-FITC (M1/70; Biolegend, UK), Ly-6C-APC (AL-21; BD Biosciences, UK), Ly-6G-PB (1A8; Biolegend, UK). Cells were collected by centrifugation, resuspended in 100 µl of neutral buffered 10% formalin (Sigma-Aldrich, Dorset, UK) and analysed using a BD LSR Fortessa cell analyser (BD Bioscience, Oxford, UK) with manual compensation for each antibody. Supplementary Fig. 3 shows the gating strategy used to identify different classes of leukocytes. Data analysis was performed using FlowJo software v8.2 (TreeStar, USA). To determine the absolute numbers of cells, 5 × 10^4^ fluorescent flow-check fluorospheres (Beckman and Coulter, UK) were added to each sample prior to analysis. Forward and side scatter were used to distinguish cell and fluorospheres populations and cell numbers were calculated from the ratio of cells to fluorospheres in each sample.

### 2.5 Measurement of plasma steroids using LC-MS/MS

Plasma levels of corticosterone and 11-dehydrocorticosterone were quantified by liquid chromatography-tandem mass spectrometry (LC-MS/MS) (Verma et al., xxxx). Briefly, plasma samples (150 μl) were enriched with epi-corticosterone (2.5 ng/sample) as an internal standard and extracted using chloroform (10:1; v/v) by briefly vortexing and discarding the upper (aqueous) layer. The organic phase was evaporated to dryness under oxygen free nitrogen at 60 °C and resuspended in water-acetonitrile (70 μl, 70:30, v/v). The resuspended steroid sample (30 µl) was injected onto a Waters Acquity™ UPLC system (Macclesfield, UK), chromatographically separated and passed into a Sciex QTRAP 5500 triple quadrupole mass spectrometer (Warrington, UK) for mass analysis and detection. The Waters Acquity™ UPLC system was fitted with an ACE ExcelC18-AR column (150 × 2.1 mm, 2 μm) protected by a Kinetex KrudKatcher® (Phenomenex, UK). The plasma steroids were separated at 40 °C using mobile phases; 0.1% formic acid in water (A), 0.1% formic acid in acetonitrile (B) with a flow rate of 0.5 mL/min. Gradient elution of 30–90% B (4–6 min with a total run time of 9 min), with epi-corticosterone, 11-dehydrocorticosterone and corticosterone eluting at 3.50, 4.95 and 5.30 min, respectively.

Following chromatographic separation the steroids were passed into a Turbospray ion source at 550 °C, with a spray voltage of 4.5 kV and an entrance potential of 10 V. Protonated molecular ions were subjected to collision induced dissociation for increased specificity and the following transitions of parent-product ion were monitored; *m*/*z* 345.1 → 121.2, 90.9 at 33 and 71 V, for 11-dehydrocorticosterone, and *m*/*z* 347.2 → 91.1, 121.1 at 69 V collision energies for the isomers corticosterone and epi-corticosterone. Data were analysed using Analyst® software v1.6.1 (SCIEX, Warrington, UK). Note that the chromatographic retention times are consistent and distinct for the isomers corticosterone and the non-endogenous internal standard epi-corticosterone.

### 2.6 11β-HSD enzyme activity assay

11β-HSD enzyme activity was measured by the conversion of [^3^H]-11-dehydrocorticosterone (A) to [^3^H]-corticosterone (B) (reductase activity) in intact peritoneal cells or by the conversion of [^3^H]-corticosterone to [^3^H]-11-dehydrocorticosterone (dehydrogenase activity) in tissue homogenates, as described previously (Coutinho et al., 2016, Low et al., 1994). Briefly, 11β-hydroxysteroid dehydrogenase activity was assayed in duplicate at 37 °C in 0.25 ml reactions containing 250 nM [^3^H]-corticosterone (TRK406-250UCI, GE Life Science Healthcare, Buckinghamshire, UK), 2 mM NADP^+^ (Sigma-Aldrich, Dorset, UK) and 190 µl homogenate in C buffer (10% glycerol, 30 mM NaCl, 1 mM EDTA, 50 mM Tris, pH7.7). Conditions were: brain, 0.5 mg protein/ml, 2 h incubation; liver, 0.2 mg protein/ml, 20 min incubation; adipose tissue, 0.2 mg protein/ml, 20 min incubation; lung, 0.5 mg protein/ml, 40 min incubation). An 11β-oxo-reductase assay was used to measure 11β-HSD1 activity in freshly isolated thioglycollate elicited peritoneal cells, lavaged 24 h after injection of 0.2 ml 10% thioglycollate, as described (Zhang et al., 2017). Peritoneal cells were resuspended in Dulbeccos’ modification of Eagle’s medium supplemented with 10% fetal bovine serum and seeded at 10^6^ cells/ml in a 24 well plate. 5 nM [^3^H]-11-dehydrocorticosterone (made as described (Zhang et al., 2017) was added and 200 µl of the culture medium was collected 2 h later. Steroids were extracted with ethyl acetate from reactions or from culture medium, separated by thin layer chromatography and quantified using a phosphorimager and tritium screen (FLA2000, Fujifilm, London, UK) with Aida image analysis software (Welford, UK).

### 2.7 RNA extraction and reverse transcription quantitative PCR analysis

RNA was extracted and quantified as described previously (Coutinho et al., 2016). Briefly, RNA was extracted using TRIzol® reagent (Thermo Fisher Scientific, Paisley UK) and reverse transcribed using SuperScript® III Reverse Transcriptase (Thermo Fisher Scientific, Paisley UK), according to the manufacturer’s instructions. Quantitative Real Time PCR (qRT-PCR) assays to quantify specific cDNAs were designed using the Roche Universal Probe Library (UPL) assay design system and were performed on a Lightcycler 480 system (Roche, West Sussex, UK). Primers (Thermo Fisher Scientific, Paisley UK) and UPL probes (Roche, West Sussex, UK) are listed in Supplementary Table 1.

### 2.8 Targeted metabolomics

Specific metabolites (listed in Supplementary Table 2) in the hippocampus were measured by BIOCRATES Life Sciences AG (Innsbruck, Austria) according to a validated experimental procedure (Ramsay et al., 2014).

### 2.9 Statistical analysis

Measurements of burrowing activity, mRNA, glucocorticoids, spleen weight and leukocyte numbers were all made on one cohort of mice (with different cohorts of mice used for each time point after vehicle or LPS). Additional and separate cohorts of mice were used for the targeted metabolomics and for measurement of 11β-HSD1 activity in tissues. Statistical analyses were performed using a Mann-Whitney test, Kruskal-Wallis test (for non-parametric data), Student’s *t*-test with Welch's correction, one way ANOVA followed by Dunnet's multiple comparison test, two-way ANOVA followed by Tukey’s multiple comparison or Fisher’s LSD test or by linear regression analysis (for parametric data), as appropriate and as described in the figure legends. A principal component analysis (PCA) was performed using the R package “FactoMineR” (Le et al., 2008). Data are means ± SEM and each value represents a single mouse. Statistical significance was set at p < .05.

## 3 Results

### 3.1 Inflammatory status and hippocampal capacity for energy metabolism is unaltered in naive *Hsd11b1^Del/Del^* mice

In the absence of an inflammatory challenge, 11β-HSD1 deficiency had no effect on markers of peripheral or brain inflammation, with undetectable *Tnfa*, *Il1b* and *Il6* mRNA in the hippocampus of *Hsd11b1^Del/Del^* and C57BL/6 control mice and no difference in circulating neutrophil and monocyte numbers between genotypes (Supplementary Fig. 4A–C). Similarly, in the hippocampus there was no difference between naïve *Hsd11b1^Del/Del^* and C57BL/6 control mice in mRNAs encoding a range of metabolic transporters and enzymes: *Slc2a1* and *Slc2a3* mRNA, encoding the GLUT-1 and GLUT-3 transporters responsible for glucose uptake in glial and neuronal cells, respectively; *Slc16a1*, *Slc16a7* and *Slc16a4* mRNA encoding, respectively, the MCT-1 and MCT-2 transporters responsible for lactate uptake in glial and neuronal cells, and the MCT-4 transporter responsible for lactate export from glial cells; key enzymes of glycolysis, the pentose phosphate pathway and mitochondrial oxidative phosphorylation (Supplementary Fig. 4D–H). These results suggest there is no inherent hippocampal inflammation in mice of either genotype, nor is the capacity for energy substrate transport and metabolism altered in the brains of naive *Hsd11b1^Del/Del^* mice. Consistent with the mRNA data, there was no difference between *Hsd11b1^Del/Del^* and C57BL/6 mice in the levels of metabolites in the hippocampus that include intermediates in glycolysis and the tricarboxylic acid (TCA) cycle (Supplementary Table 2).

### 3.2 *Hsd11b1^Del/Del^* and C57BL/6 mice show sickness behaviour following intraperitoneal injection of LPS

Inflammation leads to marked sickness behaviour (Silverman and Sternberg, 2012, Teeling et al., 2007, Tarr et al., 2012), which is considered an adaptive, beneficial, and acute response to inflammation to conserve energy for the activated immune system (Silverman and Sternberg, 2012, Hart, 1988). To test whether 11β-HSD1 influences the brain response to peripheral inflammation, *Hsd11b1^Del1/Del1^* and C57BL/6 mice were administered an intraperitoneal injection of 100 µg/kg LPS or vehicle (0.9% saline). This dose of LPS was chosen to limit the severity of the adverse effects of LPS, whilst reducing burrowing activity (an assay of sickness behaviour) and concomitantly inducing markers of inflammation in brains of treated animals, that normally resolves within 9 h (Teeling et al., 2007, Czerniawski and Guzowski, 2014).

Burrowing activity was suppressed in *Hsd11b1^Del/Del^* and C57BL/6 mice, 3 h and 6 h after LPS administration (Supplementary Fig. 5). However, 9 h after LPS administration 2 of the 8 *Hsd11b1^Del/Del^* mice had regained normal burrowing activity with the remainder showing some burrowing activity. In contrast, burrowing activity remained totally suppressed in 5 of the C57BL/6 mice at the same time point with very little activity in all but one of the remainder (Supplementary Fig. 5). These data potentially indicate quicker recovery from sickness behaviour in *Hsd11b1^Del/Del^* mice following LPS induced systemic inflammation.

Hsd11b1^Del/Del^ mice show a normal plasma corticosterone response to LPS administration, but have elevated levels of plasma 11-dehydrocorticosterone

**Fig. 1:**
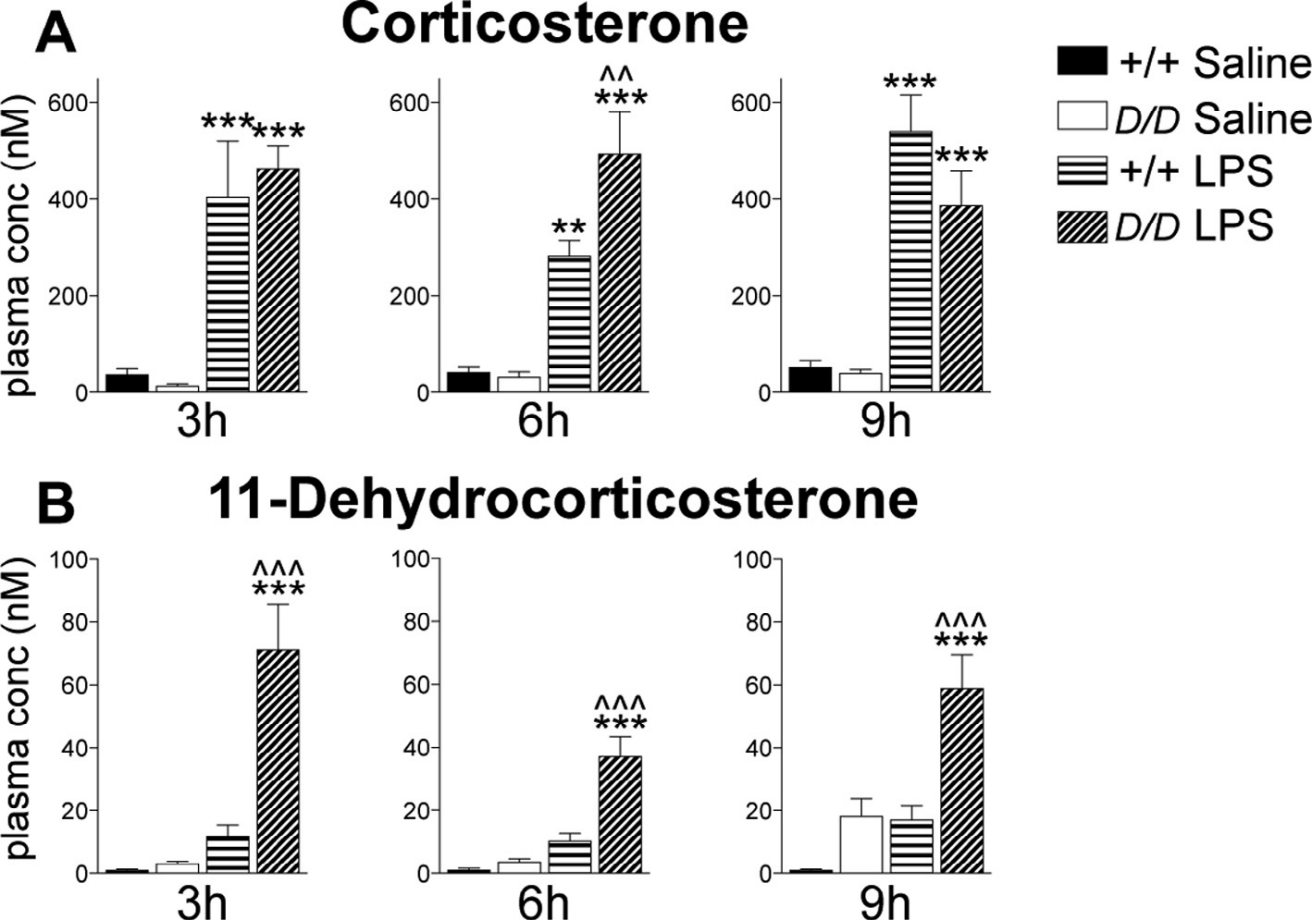
Following LPS administration, plasma corticosterone levels are similar in *Hsd11b1^Del/Del^* mice compared to controls, but 11-dehydrocorticosterone levels are elevated. *Hsd11b1^Del/Del^* and C57BL/6 mice were injected with vehicle (0.9% saline) or 100 µg/kg LPS and euthanised 3 h, 6 h or 9 h later. (A) Plasma corticosterone and (B) 11-dehydrocorticosterone levels were quantified by LC-MS/MS. Data are means ± SEM and were analysed by 2-way ANOVA followed by Tukey’s multiple comparisons tests: ^***^p < .001, ^**^p < .01 compared to the same genotype injected with saline, ^^^^^p < .001, ^^^^p < .01 compared to C57BL/6 mice with the same treatment; n = 6–9. Black bars, vehicle-treated C57BL/6 mice (+/+ Saline); white bars, vehicle-treated *Hsd11b1^Del/Del^* mice (*D/D* Saline); horizontal hatched bars, LPS-treated C57BL/6 mice (+/+ LPS); diagonal-hatched bars, LPS-treated *Hsd11b1^Del/Del^* mice (*D/D* LPS).

As expected, plasma corticosterone levels were elevated at all time points following LPS injection, with similar levels in *Hsd11b1^Del/Del^* and C57BL/6 mice (Fig. 1A). There were no genotype differences in plasma corticosterone levels in vehicle injected mice (Fig. 1A), consistent with the normal plasma corticosterone levels in a different line of 11β-HSD1 deficient mice on a C57BL/6 background (Carter et al., 2009). In contrast, for plasma 11-dehydrocorticosterone levels, 2 way-ANOVA showed a significant interaction between treatment and genotype, with markedly higher plasma 11-dehydrocorticosterone levels in LPS-injected *Hsd11b1^Del/Del^* mice, compared to saline-injected *Hsd11b1^Del/Del^* mice or to LPS-injected C57BL/6 mice (Fig. 1B).

### 3.3 Following LPS injection, circulating leukocyte numbers are similar between *Hsd11b1^Del/Del^* and C57BL/6 mice

**Fig. 2:**
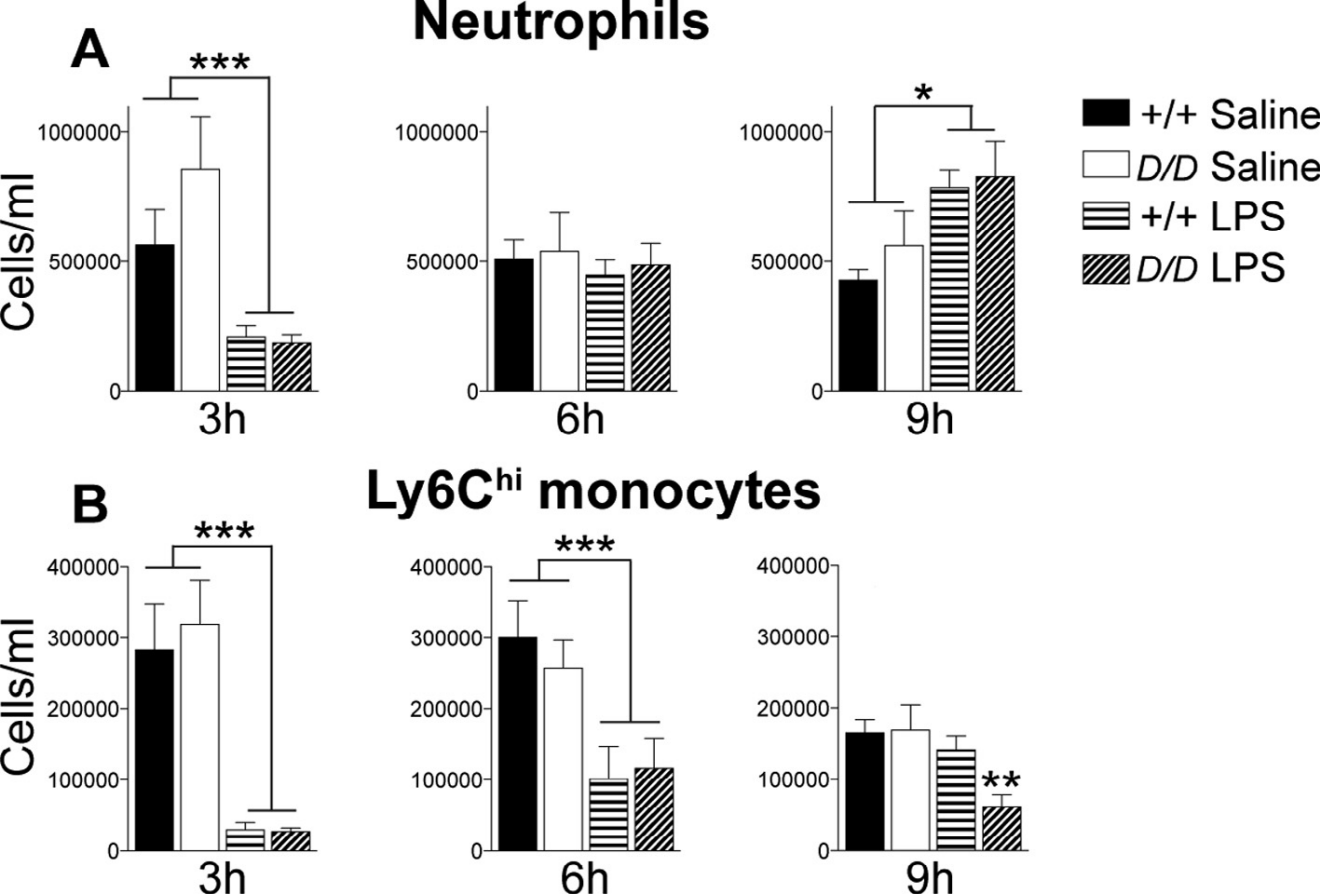
The number of circulating neutrophils and Ly6C^hi^ monocytes is similar in *Hsd11b1^Del/Del^ and C57BL/6 mice following LPS. Hsd11b1^Del/Del^* and C57BL/6 mice were injected with vehicle (0.9% saline) or 100 µg/kg LPS and euthanised 3 h, 6 h or 9 h later. (A) Neutrophils (CD45^+^CD11b^+^ Ly6G^+^Ly6C^-^ cells) and (B) Ly6C^hi^ monocytes (CD45^+^CD11b^+^ Ly6G^-^Ly6C^hi^ cells) in the blood of *Hsd11b1^Del/Del^* and C57BL/6 mice 3 h, 6 h or 9 h after vehicle or LPS injection were quantified by flow cytometry. Data are means ± SEM and were analysed by two way ANOVA followed by Fisher's LSD tests. Significant effect of treatment (saline *vs* LPS): ^***^p < .001, ^**^p < .01, ^*^p < .05; n = 5–10. Black bars, vehicle-treated C57BL/6 mice (+/+ Saline); white bars, vehicle-treated *Hsd11b1^Del/Del^* mice (*D/D* Saline); horizontal hatched bars, LPS-treated C57BL/6 mice (+/+ LPS); diagonal-hatched bars, LPS-treated *Hsd11b1^Del/Del^* mice (*D/D* LPS).

Circulating neutrophils and monocytes, key mediators of inflammation, were quantified in LPS or vehicle injected mice by flow cytometry. The numbers of circulating neutrophils (CD45^+^CD11b^+^Ly6G^+^ cells) and pro-inflammatory Ly6C^hi^ monocytes (CD45^+^CD11b^+^Ly6C^hi^Ly6G^−^ cells) were reduced 3 h after LPS injection, but with no differences between *Hsd11b1^Del/Del^* and C57BL/6 mice (Fig. 2A and B). The number of blood neutrophils recovered by 6 h after LPS injection (Fig. 2A) and by 9 h, there were more circulating neutrophils in LPS compared to vehicle-injected mice, but with no difference between genotypes (Fig. 2A). The number of Ly6C^hi^ monocytes in blood remained reduced in both *Hsd11b1^Del/Del^* and C57BL/6 mice 6 h and 9 h after LPS injection, though by 9 h the numbers of Ly6C^hi^ monocytes had largely recovered in C57BL/6 mice, whilst remaining reduced in *Hsd11b1^Del/Del^* mice (Fig. 2B). There were no differences in the number of blood neutrophils or Ly6C^hi^ monocytes between *Hsd11b1^Del/Del^* and C57BL/6 mice injected with vehicle (Fig. 2). Similarly, 3 h or 6 h following either LPS or vehicle injection there was no genotype difference in spleen weight, though spleen weight was increased by LPS in both *Hsd11b1^Del/Del^* and C57BL/6 mice (Supplementary Fig. 6). However, by 9 h after LPS, spleen weights were lower in *Hsd11b1^Del/Del^* mice than in C57BL/6 controls (Supplementary Fig. 6). Collectively, these results suggest a similar peripheral inflammatory response to this dose of LPS in *Hsd11b1^Del/Del^* and C57BL/6 mice.

### 3.4 Induction of pro-inflammatory cytokine mRNAs is attenuated in the hippocampus of *Hsd11b1^Del/Del^* mice following LPS injection

**Fig. 3:**
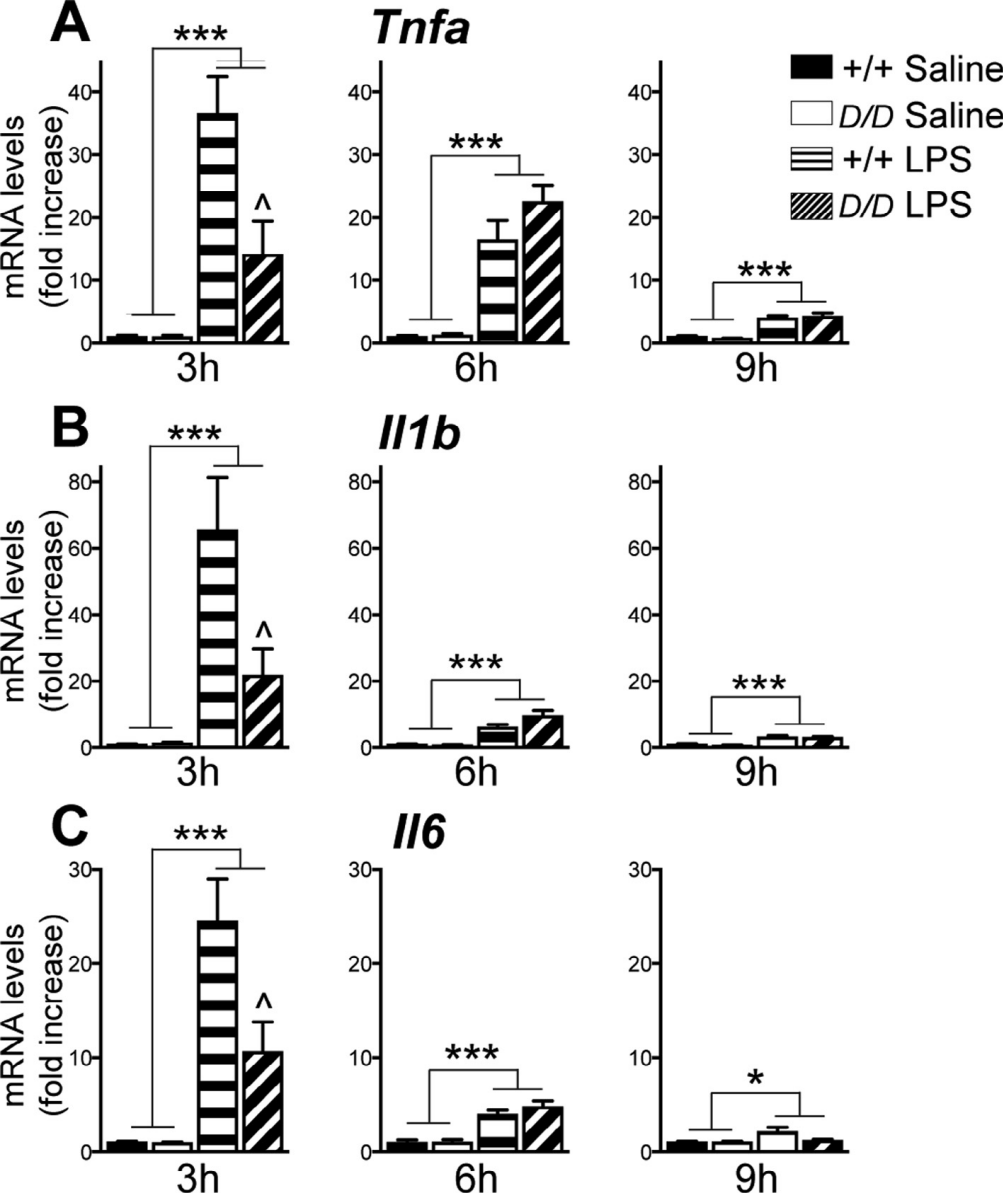
LPS induces expression of pro-inflammatory cytokine mRNAs in the hippocampus of *Hsd11b1^Del/Del^* and C57BL/6 mice. *Hsd11b1^Del/Del^* and C57BL/6 mice were injected with vehicle (0.9% saline) or 100 µg/kg LPS and euthanised 3 h, 6 h or 9 h later. Levels of mRNA in the hippocampus were quantified by RT-qPCR: (A) *Tnfa*, (B) *Il1b* and (C) *Il6*, (relative to *Hprt* and *Actb* mRNA, used as internal standard). For each time point, mRNA levels are expressed as fold change, relative to the levels in saline injected C57BL/6 mice at the 3 h time point (arbitrarily set to 1.0). Data are means ± SEM and were analysed by two way ANOVA followed by Tukey’s multiple comparisons tests. ANOVA showed a significant effect of treatment at all time points for all cytokine mRNAs (^***^p < .001, ^*^p < .05) and a significant interaction and effect of genotype for all cytokine mRNAs at 3 h. Post-hoc analysis: ^^^p < .05 compared to C57BL/6 controls with the same treatment; n = 6–8. Black bars, vehicle-treated C57BL/6 mice (+/+ Saline); white bars, vehicle-treated *Hsd11b1^Del/Del^* mice (*D/D* Saline); horizontal hatched bars, LPS-treated C57BL/6 mice (+/+ LPS); diagonal-hatched bars, LPS-treated *Hsd11b1^Del/Del^* mice (*D/D* LPS).

LPS injection induces expression of pro-inflammatory cytokines in the hippocampus (Teeling et al., 2007, Czerniawski and Guzowski, 2014). To investigate the hippocampal response to LPS in *Hsd11b1^Del/Del^* and C57BL/6 mice, levels of *Tnfa*, *Il-1b* and *Il-6* mRNA were quantified. Levels of all 3 pro-inflammatory cytokine mRNAs were elevated 3 h after LPS injection in both genotypes (Fig. 3). However, the increase at 3 h was attenuated in *Hsd11b1^Del/Del^* mice, compared to C57BL/6 controls (Fig. 3). Levels of pro-inflammatory mRNAs declined rapidly and were close to the levels in vehicle treated mice 9 h after LPS injection (Fig. 3). These results suggest that the acute pro-inflammatory response to LPS injection is attenuated in the hippocampus of *Hsd11b1^Del/Del^* mice.

### 3.5 The hippocampus of *Hsd11b1^Del/Del^* mice shows a distinct metabolic response to LPS

**Fig. 4:**
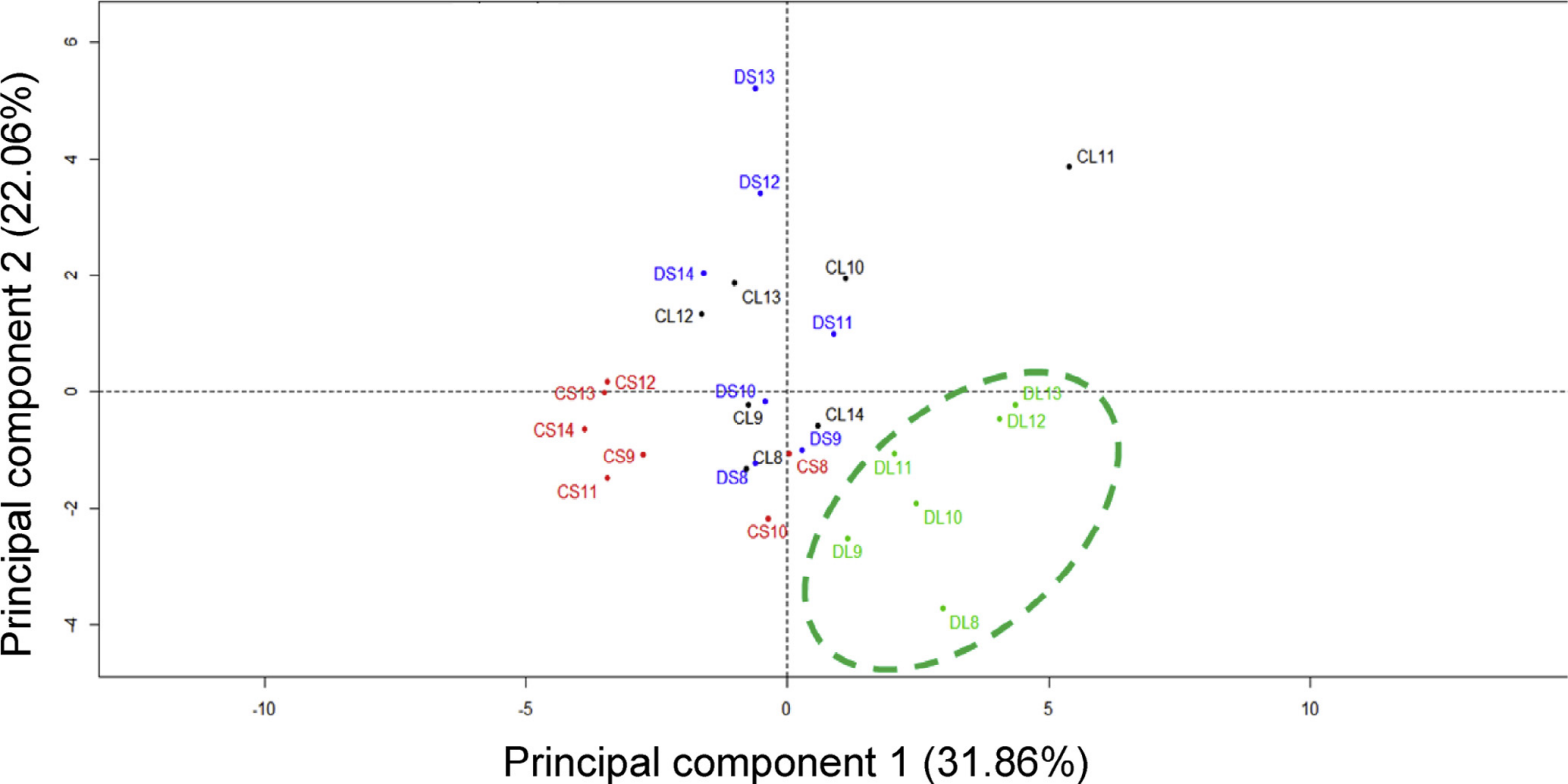
Principal component analysis shows a distinct hippocampal metabolic response in *Hsd11b1^Del/De^*^l^ mice 6 h following LPS. *Hsd11b1^Del/Del^* and C57BL/6 mice were injected with vehicle (0.9% saline) or 100 µg/kg LPS and euthanised 6 h later. Levels of mRNA encoding metabolic transporters and enzymes were quantified relative to *Hprt* and *Actb* mRNA levels by qPCR. Data were subject to principal component analysis to investigate differences between the experimental groups in the expression pattern of specific genes relevant to metabolism. Individual points represent individual mice: CL (black), C57BL/6 + LPS; CS (red), C57BL/6 + saline; DL (green) *Hsd11b1^Del/Del^* + LPS; DS (blue), *Hsd11b1^Del/Del^* + saline. A green dashed circle indicates the discrete cluster of LPS-injected *Hsd11b1^Del/Del^* mice.

To investigate whether the attenuated hippocampal pro-inflammatory response is associated with altered energy substrate uptake and/or utilisation, mRNAs encoding key metabolic enzymes and transporters were quantified in the hippocampus of *Hsd11b1^Del/Del^* and C57BL/6 mice after LPS or vehicle injection. First, to assess whether the metabolic response as a whole differed between experimental groups, principal component analysis was carried out on the mRNA data at each time point. Although principal component analysis of the 3 h and 9 h mRNA data showed different clusters for LPS or vehicle-injected mice, there were no genotype differences at these time points (Supplementary Fig. 7). However, principal component analysis of the 6 h mRNA data (cumulative variance, 54%) showed a discrete cluster for LPS-injected *Hsd11b1^Del/Del^* mice (Fig. 4). This suggests a distinct metabolic response to acute systemic inflammation in the hippocampus of *Hsd11b1^Del/Del^* mice, 6 h following LPS injection.

**Fig. 5:**
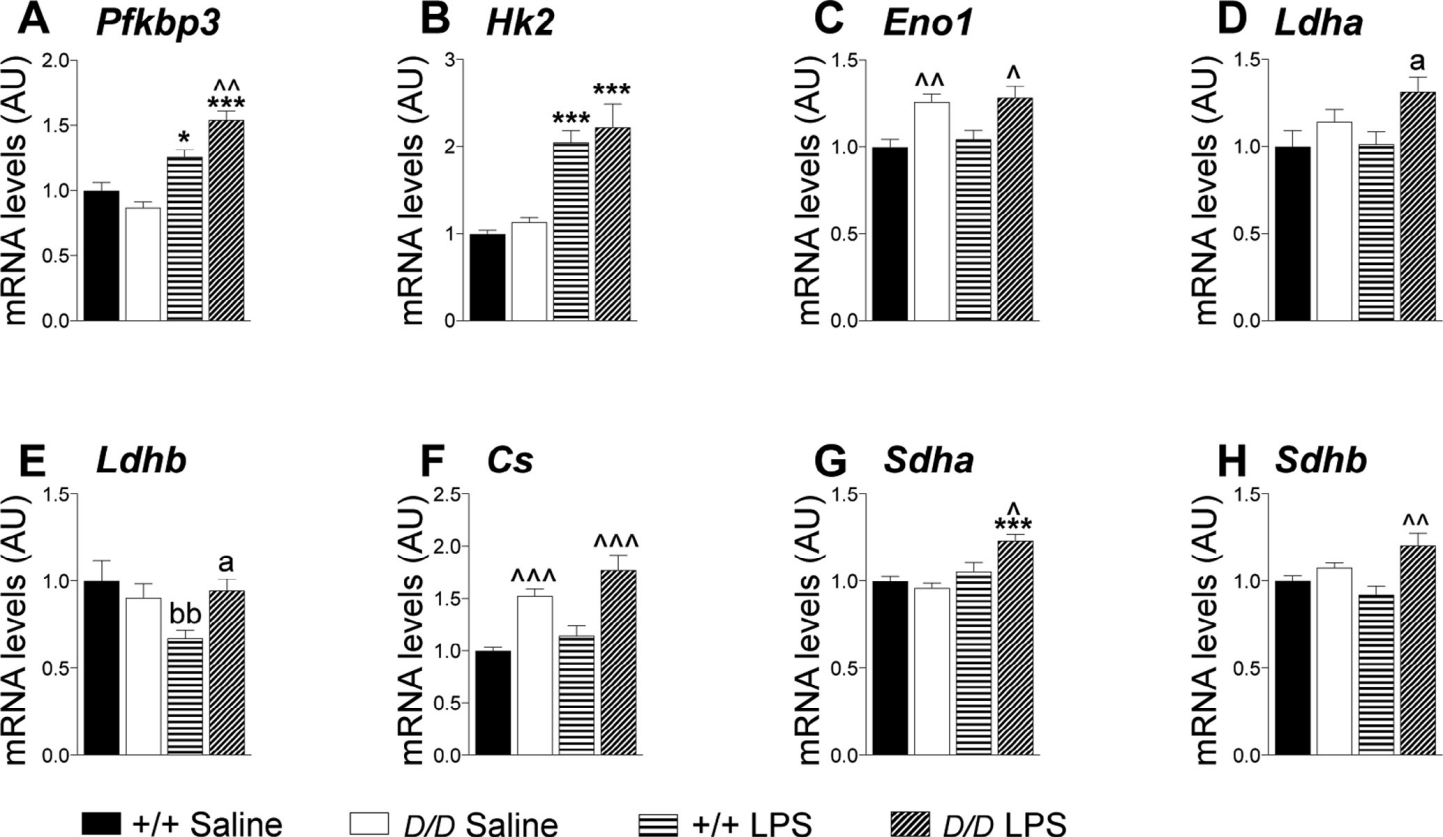
Hippocampal levels of mRNA encoding key metabolic genes suggest increased glycolysis and mitochondrial oxidation in *Hsd11b1^Del/Del^* mice, 6 h after LPS administration. *Hsd11b1^Del/Del^* and C57BL/6 mice were injected with vehicle (0.9% saline) or 100 µg/kg LPS and euthanised 6 h later. Levels of mRNAs encoding key metabolic transporters and enzymes in the hippocampus were quantified relative to the mean of *Hprt* and *Actb* mRNA levels by RT-qPCR. The levels of mRNA encoding (A–E) glycolytic enzymes and (F–H) mitochondrial oxidative phosphorylation enzymes are shown. Other enzymes and transporter mRNAs are shown in Supplementary Fig. 8. Data are means ± SEM and are expressed as fold change over levels in vehicle-injected C57BL/6 mice (arbitrarily set to 1). Data were analysed by two way ANOVA which showed a significant effect of treatment (saline *vs* LPS), p < .001 (A, B, G), p < .05 (F), a significant effect of genotype (C57BL/6 *vs Hsd11b1^Del/Del^*), p < .001 (C, F, H), p < .01 (D) and a significant interaction; p < .001 (A), p < .01 (G), p < .05 (E, H). Post-hoc tests were Tukey’s (significant effect of treatment: ^***^p < .001, ^*^p < .05 and of genotype: ^^^^^p < .001, ^^^^p < .01, ^^^p < .05) or Fisher’s LSD (significant effect of treatment: ^bb^p < .01 and of genotype: ^a^p < .05); n = 6–7. Black bars, vehicle-treated C57BL/6 mice (+/+ Saline); white bars, vehicle-treated *Hsd11b1^Del/Del^* mice (*D/D* Saline); horizontal hatched bars, LPS-treated C57BL/6 mice (+/+ LPS); diagonal-hatched bars, LPS-treated *Hsd11b1^Del/Del^* mice (*D/D* LPS).

To explore the distinct hippocampal metabolic response in *Hsd11b1^Del/Del^* mice 6 h after LPS injection, the same mRNA data set was analysed at the level of individual mRNAs and a comparison made between experimental groups (Fig. 5). To investigate the functional relevance we also measured levels of specific metabolites in the hippocampus of *Hsd11b1^Del/Del^* and C57BL/6 mice, 6 h following LPS or vehicle injection (Table 1).

**Table 1:** Hippocampal energy metabolite levels in *Hsd11b1^Del/Del^* and C57BL/6 mice 6 h post LPS or vehicle administration. *Hsd11b1^Del/Del^* (D/D) and C57BL/6 (Con) mice were injected with vehicle (0.9% saline) or 100 µg/kg LPS and euthanised 6 h later. Hippocampal energy metabolite levels were quantified by targeted metabolomics and values are expressed relative to the hippocampal tissue weights. Data are means ± SEM and were analysed by two way ANOVA (^**^p < .01, ^*^p < .05) followed by Tukey’s multiple comparisons tests: ^δ^p < .05 (*vs* same genotype with saline injection), ^##^p < .01, ^#^p < .05 (*vs* C57BL/6 mice with the same treatment), n = 6–10.

Metabolite	Saline injected (pmol/mg)		LPS injected (pmol/mg)		Interaction	Treatment	Genotype
	Con	D/D	Con	D/D			
Hexose	145.7 ± 12.1	144.5 ± 13.2	154.5 ± 15.8	119.4 ± 11.6	NS	NS	NS
Hexose phosphate	82.8 ± 18.7	89.9 ± 14.5	92.4 ± 23.2	54.2 ± 11.90	NS	NS	NS
Pyruvate + Oxaloacetate	130.7 ± 13.6	**77.2 ± 4.0**^##^	**96.7 ± 9.0^δ^**	95.9 ± 6.4	^**^	NS	^**^
Pentose phosphate	117.1 ± 12.9	118.6 ± 4.5	133.9 ± 12.0	105.2 ± 9.5	NS	NS	NS
Succ acid	157.6 ± 12.9	165.3 ± 14.0	193.6 ± 13.2	170.9 ± 15.0	NS	NS	NS
Glutamic acid	7555.0 ± 148.2	7570.0 ± 106.0	7536.0 ± 117.0	7622.2 ± 128.8	NS	NS	NS
Aspartic acid	2119.5 ± 53.0	2081.0 ± 32.2	2132.0 ± 71.2	**1941.3 ± 27.1^#^**	NS	NS	^*^
Arginine	69.4 ± 3.8	65.7 ± 2.0	71.4 ± 2.4	**61.6 ± 2.1^#^**	NS	NS	^*^

Significant differences are highlighted in bold.

Below, we focus on genes that are implicated in the Warburg effect and in regulating flux through the tricarboxylic acid (TCA) cycle. The effects of LPS treatment on the levels of other mRNAs, encoding transporters (glucose and lactate) and other enzymes involved in glycolysis or oxidative phosphorylation are described and discussed in the text that accompanies Supplementary Fig. 8.

**Fig. 6:**
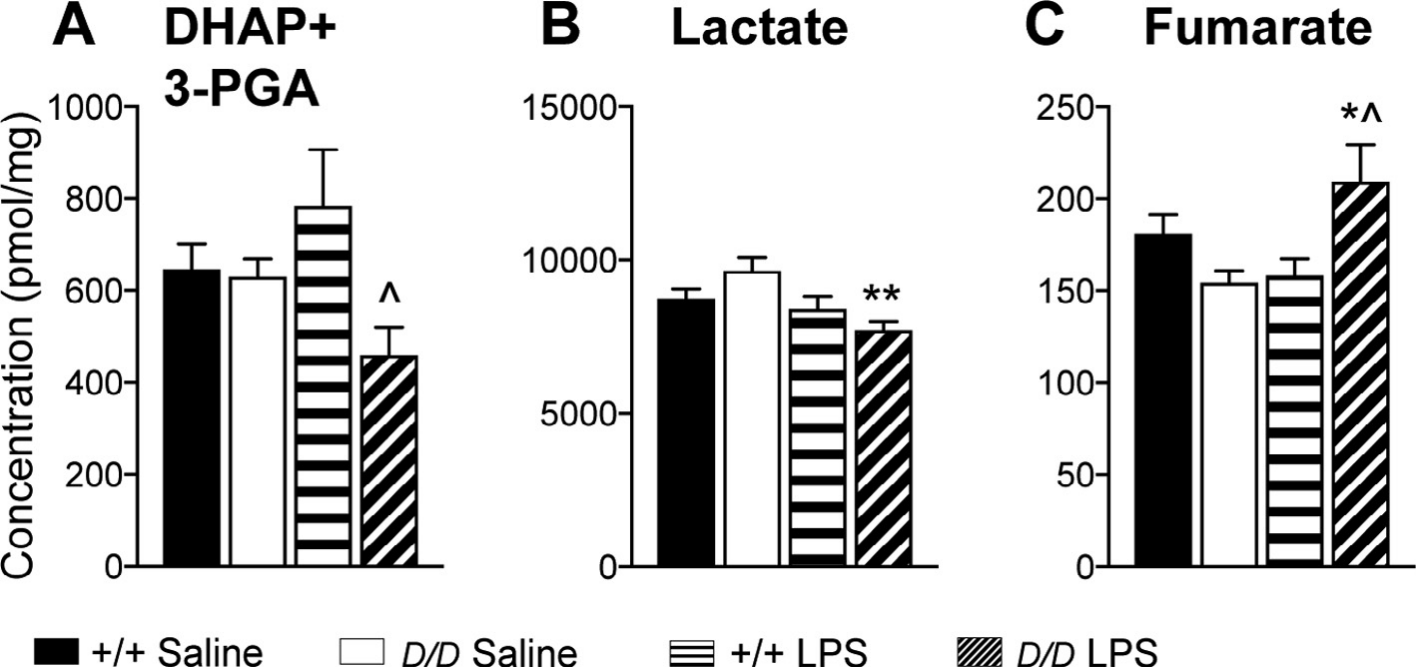
Hippocampal levels of key metabolites are altered in *Hsd11b1^Del/De^*^l^ and C57BL/6 mice 6 h post LPS. *Hsd11b1^Del/Del^* and C57BL/6 mice were injected with vehicle (0.9% saline) or 100 µg/kg LPS and euthanised 6 h later. Hippocampal levels of (A) dihydroxyacetone phosphate (DHAP) + 3-phosphoglyceraldehyde (3-PGA), (B) lactate and (C) fumarate were quantified by targeted metabolomics and values are expressed relative to the hippocampal tissue weights. Data are means ± SEM and were analysed by two way ANOVA followed by Tukey’s multiple comparisons tests: significant effect of treatment ^**^p < .01, ^*^p < .05; significant effect of genotype; ^^^p < .05 (*vs* C57BL/6 mice with the same treatment); n = 8–10. Black bars, vehicle-treated C57BL/6 mice (+/+ Saline); white bars, vehicle-treated *Hsd11b1^Del/Del^* mice (*D/D* Saline); horizontal hatched bars, LPS-treated C57BL/6 mice (+/+ LPS); diagonal-hatched bars, LPS-treated *Hsd11b1^Del/Del^* mice (*D/D* LPS).

Increased expression of *Pfkfb3* and *Hk2* is associated with aerobic glycolysis and the Warburg effect (Ando et al., 2010, Rodriguez-Prados et al., 2010, Wolf et al., 2011, De Bock et al., 2013). Consistent with a switch to aerobic glycolysis following LPS injection, levels of *Pfkfb3* and *Hk2* mRNAs were increased in both genotypes (Fig. 5A and B). Furthermore, levels of *Pfkfb3* mRNA were higher in *Hsd11b1^Del/Del^* mice than C57BL/6 controls following LPS (Fig. 5A), though *Hk2* expression did not differ between genotypes (Fig. 5B). Expression of *Eno1* is also increased in association with aerobic glycolysis (Doherty and Cleveland, 2013). Although *Eno1* mRNA levels were unaffected by LPS, they were higher in the hippocampus of *Hsd11b1^Del/Del^* mice than C57BL/6 controls (Fig. 5C). Lactate dehydrogenase (LDH) plays a key role in supporting aerobic glycolysis. By coupling pyruvate to lactate conversion with the oxidation of NADH, LDHA regenerates the NAD^+^ consumed by glyceraldehyde-3-phosphate dehydrogense (GAPDH) thereby supporting aerobic glycolysis. In contrast, LDHB preferentially converts lactate to pyruvate (Brooks, 2009, Draoui and Feron, 2011) and allows mitochondrial oxidation of lactate-derived pyruvate (Chen et al., 2016). Expression of *Ldha* is associated with the Warburg effect and with cancer whereas *Ldhb* is down-regulated in cancer (Cui et al., 2015, Leiblich et al., 2006). Levels of *Ldha* mRNA were higher in the hippocampus of *Hsd11b1^Del1/Del1^* mice than in C57BL/6 controls 6 h after LPS (Fig. 5D). In contrast, expression of *Ldhb*, was reduced in the hippocampus of C57BL/6 mice 6 h after LPS, but was unchanged in *Hsd11b1^Del1/Del1^* mice (Fig. 5E). These data support an increase in the capacity for aerobic glycolysis in *Hsd11b1^Del/Del^* mice compared to controls, following LPS injection, with increased flux through the GAPDH reaction supported by higher expression of *Ldha*. Consistent with this hypothesis, levels of 3-phosphoglyceraldehyde (the substrate of GAPDH), together with dihydroxyacetone phosphate (produced in equimolar amounts with 3-phosphoglyceraldehyde during glycolysis) were decreased in the hippocampus of *Hsd11b1^Del1/Del1^* mice 6 h after LPS, but were unaffected by LPS in control mice and did not differ between the genotypes in saline injected mice (Fig. 6A). Interestingly, levels of lactate, the end product of aerobic glycolysis, were lower in the hippocampus of *Hsd11b1^Del1/Del1^* mice 6 h after LPS compared to C57BL/6 or to saline injected mice (Fig. 6B), suggesting increased use of lactate as a substrate for further (mitochondrial) metabolism during inflammation, possibly through maintained expression of *Ldhb*.

We next looked at hippocampal expression of genes and metabolites involved in mitochondrial oxidative metabolism. Levels of *Cs* mRNA, encoding citrate synthase and a marker of mitochondrial number (Hughes et al., 2014), were increased in *Hsd11b1^Del1/Del1^* mice compared to C57BL/6 controls, irrespective of treatment (Fig. 5F). Levels of pyruvate + oxaloacetate, which together feed into the citrate synthase reaction, were lower in saline-injected *Hsd11b1^Del/Del^* mice compared to C57BL/6 (Table 1), suggesting flux through citrate synthase may be increased in *Hsd11b1^Del/Del^* mice. Although pyruvate + oxaloacetate levels were reduced by LPS in control mice, they were unaffected in *Hsd11b1^Del/Del^* mice (Table 1). Succinate dehydrogenase (SDH) is essential in both the TCA cycle and as complex II of the electron transport chain. It is an important sensor of ischemia: its substrate, succinate, is an inducer of inflammation (Chouchani et al., 2014, Tannahill et al., 2013) and accumulation of either succinate or its product, fumarate can cause pseudo-hypoxia (Selak et al., 2005, Isaacs et al., 2005). Moreover, SDH is the determinant of the reserve respiratory capacity that allows cells to increase ATP under conditions when energy demand exceeds supply (Pfleger et al., 2015). Expression of *Sdha* and *Sdhb* (encoding the A and B subunits of succinate dehydrogenase, respectively) showed a significant interaction between genotype and treatment so that 6 h after LPS injection, levels were higher in hippocampus of *Hsd11b1^Del1/Del1^* mice compared to C57BL/6 controls (Fig. 5G and H). Consistent with the mRNA data, two-way ANOVA showed a significant interaction for fumarate levels, which were increased in *Hsd11b1^Del/Del^* compared to C57BL/6 mice following LPS injection (Fig. 6C). Succinate levels were unaltered (Table 1). Collectively, these results suggest an increase in mitochondrial oxidative metabolism and reserve respiratory capacity in *Hsd11b1^Del/Del^* compared to C57BL/6 mice, following LPS injection.

### 3.6 Hippocampal *Hsd11b1* mRNA levels are down-regulated by systemic inflammation

Pro-inflammatory cytokines and raised plasma glucocorticoid levels increase *Hsd11b1* expression in most tissues (Chapman et al., 2013, Morgan et al., 2014). We therefore measured levels of *Hsd11b1* mRNA in the hippocampus of LPS injected C57BL/6 mice. Steady-state *Hsd11b1* mRNA levels in the hippocampus were unaffected 3 h and 6 h after LPS injection but were reduced by 9 h (Supplementary Fig. 9A). Moreover, we observed down-regulation of hippocampal *Hsd11b1* mRNA levels in two distinct models of inflammation (Supplementary Fig. 9B), suggesting that down-regulation of *Hsd11b1* in the hippocampus might be a general response to systemic inflammation.

## 4 Discussion

11β-HSD1-deficient mice show an attenuated hippocampal pro-inflammatory cytokine response to peripheral administration of a moderate dose of LPS. This occurs despite no difference in the number of circulating inflammatory cells or in the plasma corticosterone response to LPS. Previous studies in a different line of 11β-HSD1 deficient mice to that used here showed a greater peripheral inflammatory response, compared to controls (Zhang and Daynes, 2007, Coutinho et al., 2012). Zhang and Daynes (2007) reported a modest increase in the plasma cytokine response to a much higher dose of LPS than used here, but did not measure the number of circulating cells Similarly, circulating cells were not measured in our previous study in which we showed greater recruitment of inflammatory cells to the serous cavities in *Hsd11b1^−/−^* mice in models of inflammation that (unlike the response to LPS) are dominated by an early neutrophil mediated response (Coutinho et al., 2012). This suggests that protection from a pro-inflammatory hippocampal response to peripheral administration of LPS is mediated at a tissue-specific level, within the brain. Local activation of GR within the hippocampus has been implicated in priming of microglia and potentiation of neuroinflammatory responses to LPS (Frank et al., 2010, Frank et al., 2012, Barrientos et al., 2015). Whether the microglia are involved in the effects we see here is an interesting question for the future. We note, however, that we have not formally ruled out blood contamination as a source of the mRNA changes we see in hippocampus (brains were not perfused at sacrifice, to remove blood contamination. Cytokine mRNAs are low abundance in normal brain and are the mRNAs most likely to be affected by blood contamination (given that the blood volume in the hippocampus is tiny and the contribution of leukocyte RNA to total hippocampal levels of mRNAs encoding enzymes in metabolic pathways will be insignificant). A previous study (Gabellec et al., 1995), using the same dose and a similar time course of LPS administration as used here, directly compared cytokine mRNAs in saline perfused and not-perfused brains, with identical results for mRNAs encoding IL-1α, IL-1 β and IL-1ra. This suggests it is extremely unlikely that blood contamination could account for the increase in pro-inflammatory cytokine levels we find in hippocampus after LPS.

The attenuated pro-inflammatory cytokine response in the hippocampus of *Hsd11b1^Del/Del^* mice is associated with an altered metabolic response that is particularly marked 6 h after injection of LPS. This is dominated by an apparent “Warburg” effect in which expression of key glycolytic enzymes that are typically induced by hypoxia or in cancer, is increased. These include *Pfkfb3* and *Ldha*, both of which are increased by LPS to a greater extent in *Hsd11b1^Del/Del^* mice than controls. PFKFB-3 generates fructose 2,6-bis phosphate, a potent activator of PFK1 activity, thereby accelerating flux through a major rate-limiting check-point of the glycolytic pathway. Likewise, by regenerating NAD^+^ concomitant with lactate production from pyruvate as the last step of glycolysis, LDHA supports the flux through GAPDH and thus, glycolysis. An increase in flux through glycolysis in the hippocampus of LPS injected *Hsd11b1^Del/Del^* mice is supported by the measurement of glycolytic metabolites, with reduced levels of 3-phosphoglyceraldehyde and dihydroxyacetone phosphate, that are produced downstream of PFK1 and which feed into GAPDH. However, the metabolite measurements only provide a snapshot at any one time, and metabolic flux analysis using labelled tracers is required to confirm that glycolytic flux is indeed increased in *Hsd11b1^Del/Del^* mice following LPS. Nevertheless, these data strongly support an increase in glycolysis in the brains of *Hsd11b1^Del/Del^* mice in response to inflammation. Interestingly, loss of *HSD11B1* expression has been reported to enhance glycolysis in liver cancer (Liu et al., 2016). PET imaging of ^18^F-FDG uptake showed that over-expression of 11β-HSD1 in hepatocarcinoma cells reduced glucose uptake and glycolysis *in vitro,* and, when implanted in mice, reduced intrahepatic metastasis of hepatocarcinoma, angiogenesis and tumour growth *in vivo* (Liu et al., 2016).

The possibility of an increased glycolytic flux is relevant to several aspects of the phenotype of 11β-HSD1 deficiency or inhibition. In addition to facilitating tumour growth, glycolysis plays a key role in tissue repair following injury. Glycolysis is of crucial importance in fuelling lymphocyte activation (Pearce et al., 2013) and is also fundamental to the process of angiogenesis and vessel branching. Loss of PFKFB3 in endothelial cells impairs angiogenesis through reduced vessel sprouting and endothelial cell proliferation (De Bock et al., 2013). Importantly, deficiency in, or inhibition of 11β-HSD1 is associated with increased angiogenesis in a variety of contexts: in a sponge model of inflammatory angiogenesis, during cutaneous wound healing, in the healing heart following coronary artery ligation and in hypoxic adipose tissue during obesity (Small et al., 2005, McSweeney et al., 2010, Michailidou et al., 2012). Our data here suggest that 11β-HSD1-deficiency or inhibition increases the induction of *Pfkfb3* expression that occurs during the Warburg effect. The mechanism by which this occurs is unknown, but could result from the inflammatory response seen here in brain, and associated with tissue injury and repair. Increased *Ldha* expression is also a hallmark of aerobic glycolysis. In the normal brain, *Ldha* is strongly expressed in the hippocampus (Laughton et al., 2000), and has been suggested to play a role in synapse formation and growth (Goyal et al., 2014). Similarly, aerobic glycolysis may be protective in mouse models of Alzheimer’s disease (Newington et al., 2012). On the other hand, it has been suggested that high brain lactate levels are a hallmark of aging, due to mitochondrial failure and consequent reliance on glycolysis (Ross et al., 2010). However, we found no evidence of mitochondrial dysfunction in *Hsd11b1^Del/Del^* mice (*Cs* and *Cox4i1* were increased in both saline and LPS injected *Hsd11b1^Del/Del^* mice) and indeed, hippocampal lactate levels were lower after LPS than in control mice. Whether a greater propensity for aerobic glycolysis contributes to the neuroprotective effect of 11β-HSD1 deficiency/inhibition during cognitive aging (Yau et al., 2015, Yau et al., 2001) or in mouse models of Alzheimer’s disease (Sooy et al., 2015) remains to be determined.

Lactate represents an important metabolic fuel in brain. Although we find evidence for increased brain glycolysis in *Hsd11b1^Del/Del^* mice following LPS, there is no evidence for a corresponding increase in lactate levels. This raises the possibility that lactate is used by an intracellular lactate shuttle to a greater extent in *Hsd11b1^Del/Del^* mice than in controls after LPS. In support of this notion, *Ldhb* expression is maintained in LPS injected *Hsd11b1^Del/Del^* mice (though decreased in controls). This is consistent with a greater level of lactate to pyruvate conversion as a fuel for mitochondrial oxidation (Brooks, 2009, Draoui and Feron, 2011, Chen et al., 2016). Further support for this idea comes from the higher level of *Cs* expression and lower levels of pyruvate + oxaloacetate (substrates for the TCA cycle) in *Hsd11b1^Del/Del^* mice. Intriguingly, we found an interaction between genotype and treatment in the expression of the genes encoding two of the subunits of SDH/complex II, with higher expression of *Sdha* and *Sdhb* in LPS injected *Hsd11b1^Del/Del^* mice, as well as a similar interaction in levels of fumarate, the product of SDH. Accumulation of fumarate can cause pseudo-hypoxia by increasing HIF-1α stability (Isaacs et al., 2005, Sullivan et al., 2013). This plausibly accounts for the increase in the HIF-1 α target genes involved in aerobic glycolysis that also showed the interaction between genotype and treatment in LPS injected *Hsd11b1^Del/Del^* mice.

Whilst hypoxic signalling *in vitro* is not significantly altered with 11β-HSD1 deficiency (Michailidou et al., 2012), the metabolic alterations and gene expression changes we see in brain are all suggestive of an increased propensity to induce HIF-1 α and thus hypoxic response genes in the brain of *Hsd11b1^Del/Del^* mice during inflammation. The underlying mechanisms merit further investigation. We did not find succinate to be increased but succinate accumulation is a universal signature of tissue ischaemia that, during ischaemia–reperfusion, leads to excessive mitochondrial ROS production and ischemia–reperfusion injury, including in brain (Chouchani et al., 2014). Increased SDH/complex II activity might reduce ROS damage during systemic inflammation (Chouchani et al., 2014, Guzy et al., 2008). Another mechanism by which elevated SDH expression may be neuroprotective is through determination of mitochondrial reserve respiratory capacity. Reserve capacity is a major factor in cell survival following neurotoxic (Yadava and Nicholls, 2007) or ischemic (Pfleger et al., 2015) insult. Interestingly in this respect, studies using carbenoxolone (a non-selective inhibitor of 11β-HSD enzymes) and BVT-2733 (a selective inhibitor of 11β-HSD1) found they provided neuroprotection against ischemic brain injury in an acute setting (Beraki et al., 2013). Whether this latter is mediated at SDH/complex II is unknown, but our data suggest that an increase in flux through SDH should be investigated as a potential mechanism underlying this neuroprotective effect of 11β-HSD1 inhibition.

11-DHC levels are markedly elevated in *Hsd11b1^Del/Del^* mice when activity of the HPA axis is increased: either during the normal diurnal rise (in saline injected mice) or in response to LPS. This is an indication of the level of substrate normally available to 11β-HSD1. However, the fate of corticosterone generated from 11-DHC in C57BL/6 mice is unclear: plasma levels are similar in *Hsd11b1^Del/Del^* and control mice. Mass spectrometry imaging of the mouse brain has shown a reduction in the intracellular corticosterone/11-DHC ratio with 11β-HSD1 inhibition/deficiency including in the hippocampus, chiefly because of increased 11-DHC levels (Cobice et al., 2013). 11β-HSD1-deficient mice are protected from the metabolic effects of corticosterone excess (Morgan et al., 2014) as well as the adverse cognitive effects of aging or stress – both activators of the HPA axis. During stress or in aged mice, intrahippocampal corticosterone levels are reduced with 11β-HSD1-deficiency, despite similar plasma corticosterone levels (Yau et al., 2015). Our data here suggest that during peripheral inflammation, a reduction in intrahippocampal corticosterone levels is associated with an attenuated pro-inflammatory and a rapid metabolic response. Whether these are mediated via the higher affinity MR or via GR is currently unclear (Yau et al., 2011), and indeed, they may share a common mechanism or be distinct. Nevertheless, the altered brain inflammatory response associated with increased glycolysis, mitochondrial respiration and induction of HIF-1 α target genes suggests this may be a neuroprotective mechanism during acute inflammation. Whether a similar mechanism underlies the neuroprotective effect of 11β-HSD1-deficiency/inhibition from accumulative damage caused by life-time exposure to stresses that increase HPA axis activity is an important question for the future.
