# Supplementary material for: 11β-hydroxysteroid dehydrogenase-1 deficiency alters brain energy metabolism in acute systemic inflammation
Source: Brain Behav Immun. 2018 Mar;69:223–34. doi: 10.1016/j.bbi.2017.11.015 (PMC5871395; doi:10.1016/j.bbi.2017.11.015)
Supplement: Supplementary data 1 [file mmc1.docx]

**VERMA ET AL, SUPPLEMENTARY INFORMATION**

| Gene | Primer sequence | UPL probe number | Reference |
| --- | --- | --- | --- |
| *Actb* | F - CTAAGGCCAACCGTGAAAAG  R - ACCAGAGGCATACAGGGACA | 64 | (1) |
| *18S* | F - CTCAACACGGGAAACCTCAC  R - CGCTCCACCAACTAAGAACG | 77 | (1) |
| *Gapdh* | F – GGTTCCTATAAATACGCACTGC  R - CCATTTTGTCTACGGGACGA | 52 | (1) |
| *Hprt* | F – TCCTCCTCAGACCGCTTTT  R - CCTGGTTCATCATCGCTAATC | 95 | (2) |
| *Hsd11b1* | F - GGAGCCCATGTGGTATTGAC  R - TTCAAGGCAGCGAGACACTA | 69 | (3) |
| *Slc16a1* | F - ATGCTGCCCTGTCCTCCT  R - CCACAAGCCCAGTACGTGTAT | 49 | This work |
| *Slc16a7* | F - TCGTGGAGTGTTGTCCAGTT  R - TCCGCTGGCTATGTACAGGT | 20 | This work |
| *Slc16a4* | F - AAGCATTATCCAGATCTACCTCAC  R - GGCGACGCTTGTTGAAGTAT | 11 | This work |
| *Ldha* | F - AACCTTAGGCGGGTGCAT  R - GGAAGACATCCTCATTGATTCC | 31 | This work |
| *Ldhb* | F - GAAAATTGTGGCCGATAAAGA  R - TCTCTGCACCAGGTTGAGC | 51 | This work |
| *Hk1* | F - TCCCAGATGGGACTGAGC  R - GGACTCGGAAATTCGTTCCT | 72 | (3) |
| *Hk2* | F - CAACTCCGGATGGGACAG R - CACACGGAAGTTGGTTCCTC | 21 | This work |
| *Pfkfb3* | F – ATGGAATTAGAGCGCCAAGA  R - CATTTCAGGTATGGCATCTCC | 64 | This work |
| *Pfk1* | F – CACAGGATTGTGGAGATCGTAG  R - CACAGGATTGTGGAGATCGTAG | 9 | This work |
| *Slc2a1* | F – ATGGATCCCAGCAGCAAG  R - CCAGTGTTATAGCCGAACTGC | 52 | This work |
| *Slc2a3* | F – GCATTTGGCACACTAAACCA  R - GCCCAGAATAAAGTCCAAACC | 105 | This work |
| *Tnfa* | F – TCTTCTCATTCCTGCTTGTGG  R - GGTCTGGGCCATAGAACTGA | 49 | (4) |
| *Il1b* | F – TGTAATGAAAGACGGCACACC  R - TCTTCTTTGGGTATTGCTTGG | 78 | (4) |
| *Cs* | F - CCCAACGTAGACGCTCACA  R - TTTCCAGGGGGAAGCCTA | 50 | This work |
| *Eno1* | F - GAGGACACTTTCATCGCAGAC  R - CCAGCTCTTCCTCAATTCTGA | 77 | This work |
| *Eno2* | F - AACAGCGTTACTTAGGCAAAGG  R - CCACCACGGAGATACCTGAG | 18 | This work |
| *Sdha* | F - TGTTCAGTTCCACCCCACA  R - TCTCCACGACACCCTTCTG | 71 | This work |
| *Sdhb* | F - CTGGTGGAACGGAGACAAGT  R - GCGTTCCTCTGTGAAGTCGT | 42 | This work |
| *Cox4i1* | F - TCACTGCGCTCGTTCTGAT  R - CGATCGAAAGTATGAGGGATG | 7 | This work |
| *G6pdx* | F - ACGACATCCGAAAGCAGAGT  R - CATAGGAATTACGGGCAAAGA | 78 | This work |
| *Il6* | F - GCTACCAAACTGGATATAATCAGGA  R – CCAGGTAGCTATGGTACTCCAGAA | 6 | (2) |

***Supplementary Table 1. Primer-probe sets used for qPCR*.**

Assays were designed using the Roche Universal Probe Library design centre, <https://lifescience.roche.com/en_gb/articles/Universal-ProbeLibrary-System-Assay-Design.html>.

| Metabolite | C57BL/6  (pmol/mg) | *Hsd11b1^Del/Del^*  (pmol/mg) | p value |
| --- | --- | --- | --- |
| Hexose | 193.9 ± 12.8 | 170.4 ± 18.3 | NS |
| Hexose phosphate | 133.9 ± 17.8 | 103.6 ± 15.8 | NS |
| Dihydroxyacetone phosphate + 3-Phosphoglyceraldehyde | 798.0 ± 61.0 | 721.8 ± 90.3 | NS |
| Pyruvate + Oxaloacetate | 96.4 ± 9.8 | 114.9 ± 11.4 | NS |
| Lactate | 10140 ± 294 | 10950 ± 684 | NS |
| Pentose phosphate | 112.3 ± 8.0 | 124.1 ± 17.4 | NS |
| Succinic acid | 103.5 ± 18.7 | 140.6 ± 10.9 | NS |
| Fumaric acid | 171.2 ± 7.5 | 173.3 ± 8.0 | NS |
| Glutamic acid | 6680.0 ± 128.7 | 7000.0 ± 228.9 | NS |
| Aspartic acid | 2378.0 ± 64.6 | 2307.0 ± 114.4 | NS |
| Arginine | 83.6 ± 3.9 | 89.7 ± 2.0 | NS |
| 3’-5’-cyclic Adenosine | ND | ND |  |
| α-ketoglutaric acid | ND | ND |  |

***Supplementary Table 2. Metabolite levels in the hippocampus of naïve Hsd11b1^Del/Del^ and C57BL/6 control mice.***

Levels of specific metabolites in the hippocampus of naïve *Hsd11b1^Del/Del^* and C57BL/6 control mice were measured by BIOCRATES Life Sciences AG (Innsbruck, Austria) as described in Materials and Methods and are expressed in pmol/mg of hippocampal tissue. Data are means ± SEM and were analysed by students *t*-test, n=8. NS: not significant (p>0.05), ND: not detected.


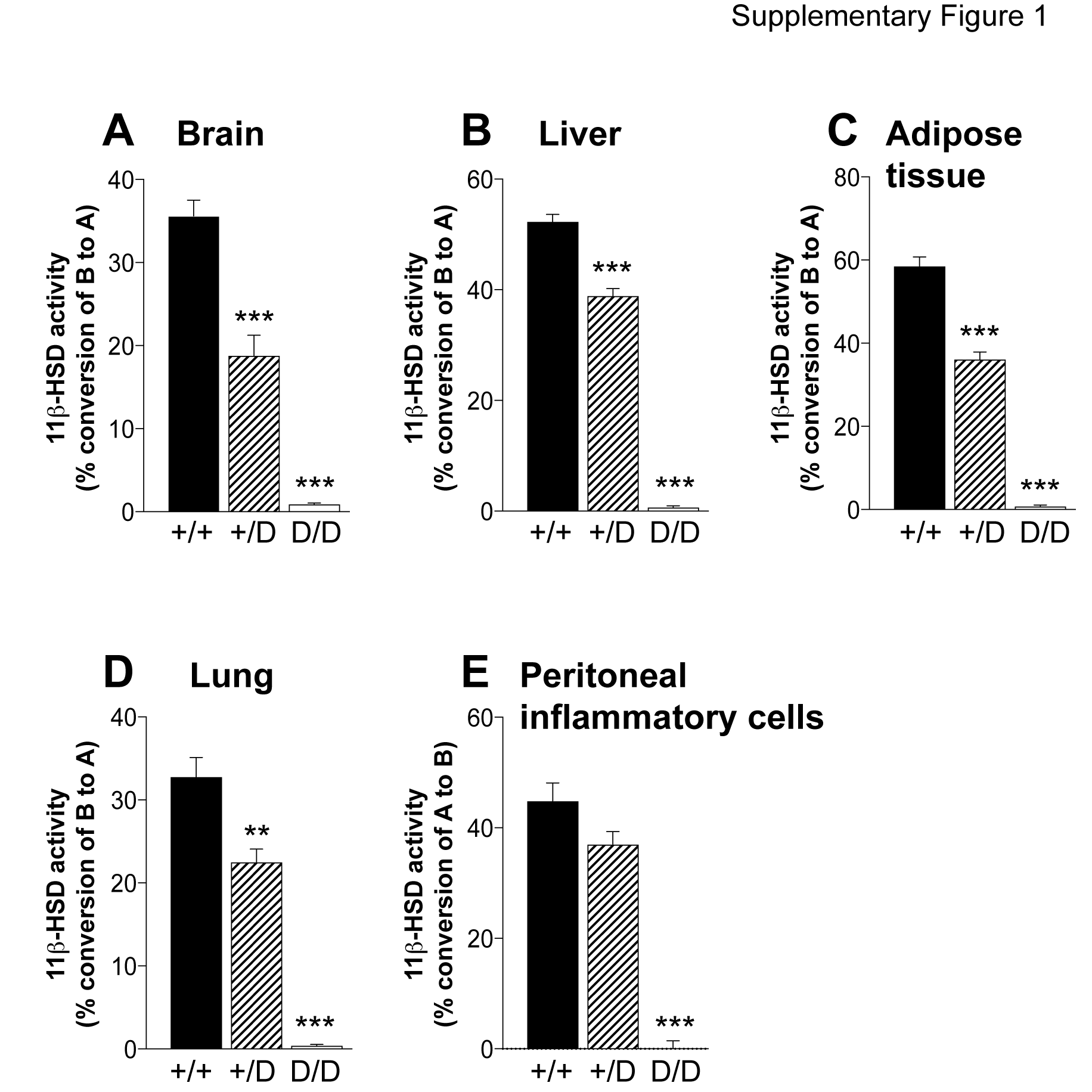


***Supplementary Figure 1. Hsd11b1^Del/Del^ mice lack 11β-HSD activity in brain, lung, liver, adipose tissue and peritoneal inflammatory cells.***

Tissue homogenates from C57BL/6 controls (+/+, black bars), heterozygous *Hsd11b1^+/Del^* (+/D, diagonal hatched bars) and homozygous *Hsd11b1^Del/Del^* (D/D, white bars) mice were assayed for 11β-HSD activity: (A) brain, (B) liver, (C) adipose tissue and (D) lung. Activity is expressed as % conversion of corticosterone [B] to 11-dehydrocorticosterone [A]. Conditions are described in Materials and Methods and were chosen to give less than 50% conversion in homogenates from heterozygous mice (+/D). (E) An 11β-reductase assay was carried out on peritoneal cells lavaged 24h following intra-peritoneal injection of 0.2ml 10% thioglycollate. Data are means ± SEM and were analysed by one way ANOVA, followed by Dunnett’s multiple comparison test: ^***^p<0.001 ^**^p<0.01, compared to C57BL/6 controls. (A-D) n=6-10, (E) n=5-7 mice.

*
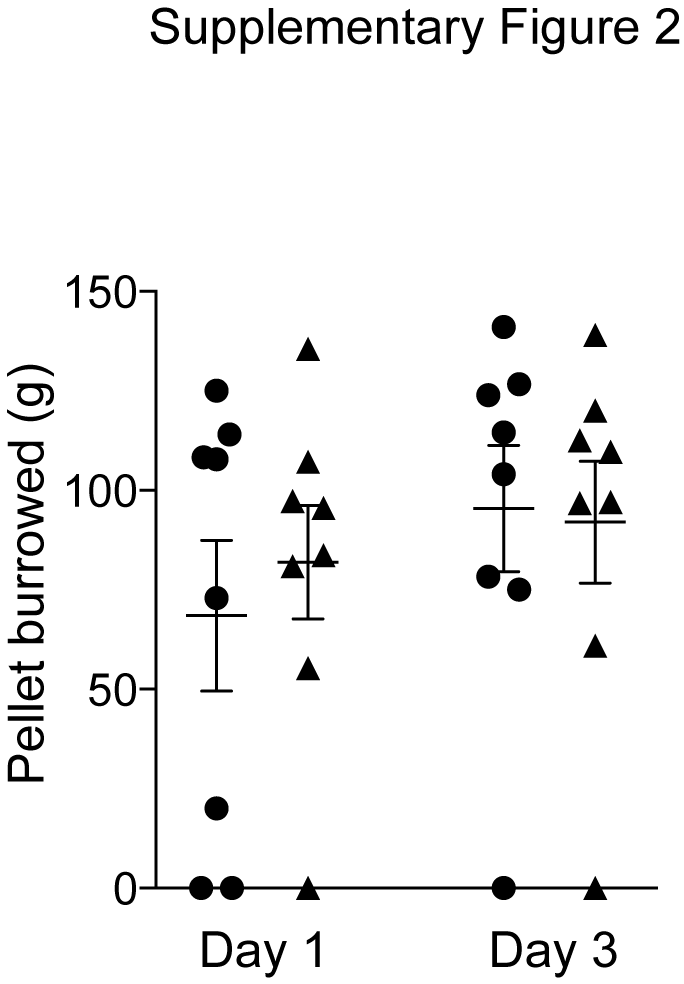
*

***Supplementary Figure 2. Baseline burrowing activity did not differ between Hsd11b1^Del/Del^ and C57BL/6 control mice.***

Sickness behaviour was assayed using a food burrowing test. A tube sealed at one end and elevated at the other was filled with 150g food pellets and placed in the cage with singly housed mice. Following overnight habituation, baseline burrowing activity was measured in a 2h period on day 1 and day 3. The weight of food pellets removed from the tube (burrowing activity) in baseline tests did not differ between C57BL/6 (circles) and *Hsd11b1^Del/Del^* mice (triangles). Values show individual mice, with horizontal bars indicating the medians ± interquartile ranges. Data were analysed by Kruskal-Wallis test, with pair-wise comparison by Mann-Whitney test, n=7-8.

*
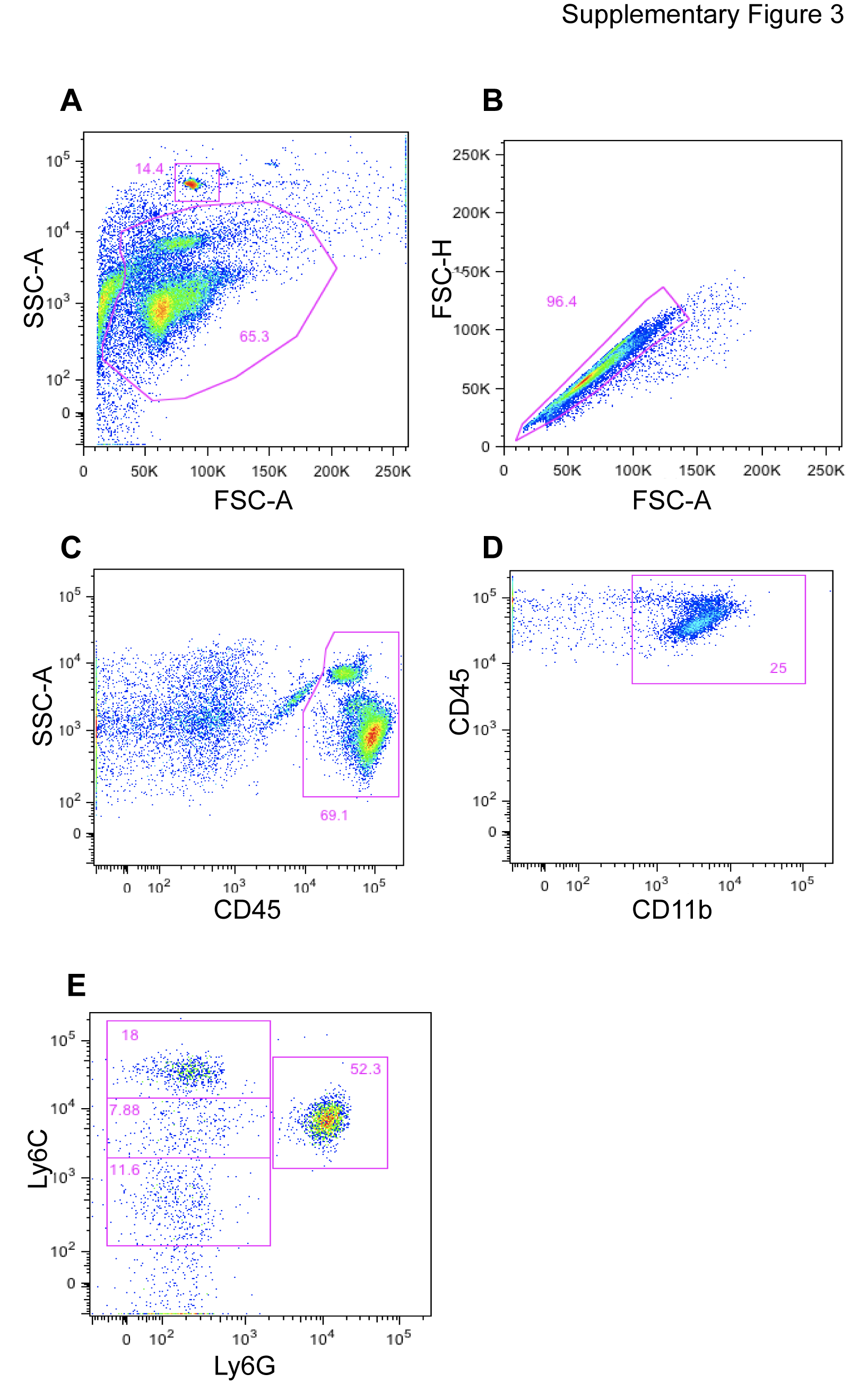
*

***Supplementary Figure3. Gating strategy for quantifying different classes of circulating leukocytes in whole blood.***

(A) Cells and flow check beads were identified using forward (FSC) and side scatter (SSC) gating. (B) Within the cell gate, singlet cells were identified using FSC. (C) Total leukocytes (CD45^+^ cells), (D) total myeloid cells (CD45^+^CD11b^+^), (E) monocyte sub-populations (Ly6C^hi^, Ly6C^med^ and Ly6C^lo^) and polymorphonuclear leukocytes consisting mainly of neutrophils (Ly6G^+^) were identified and quantified as described in the text.

*
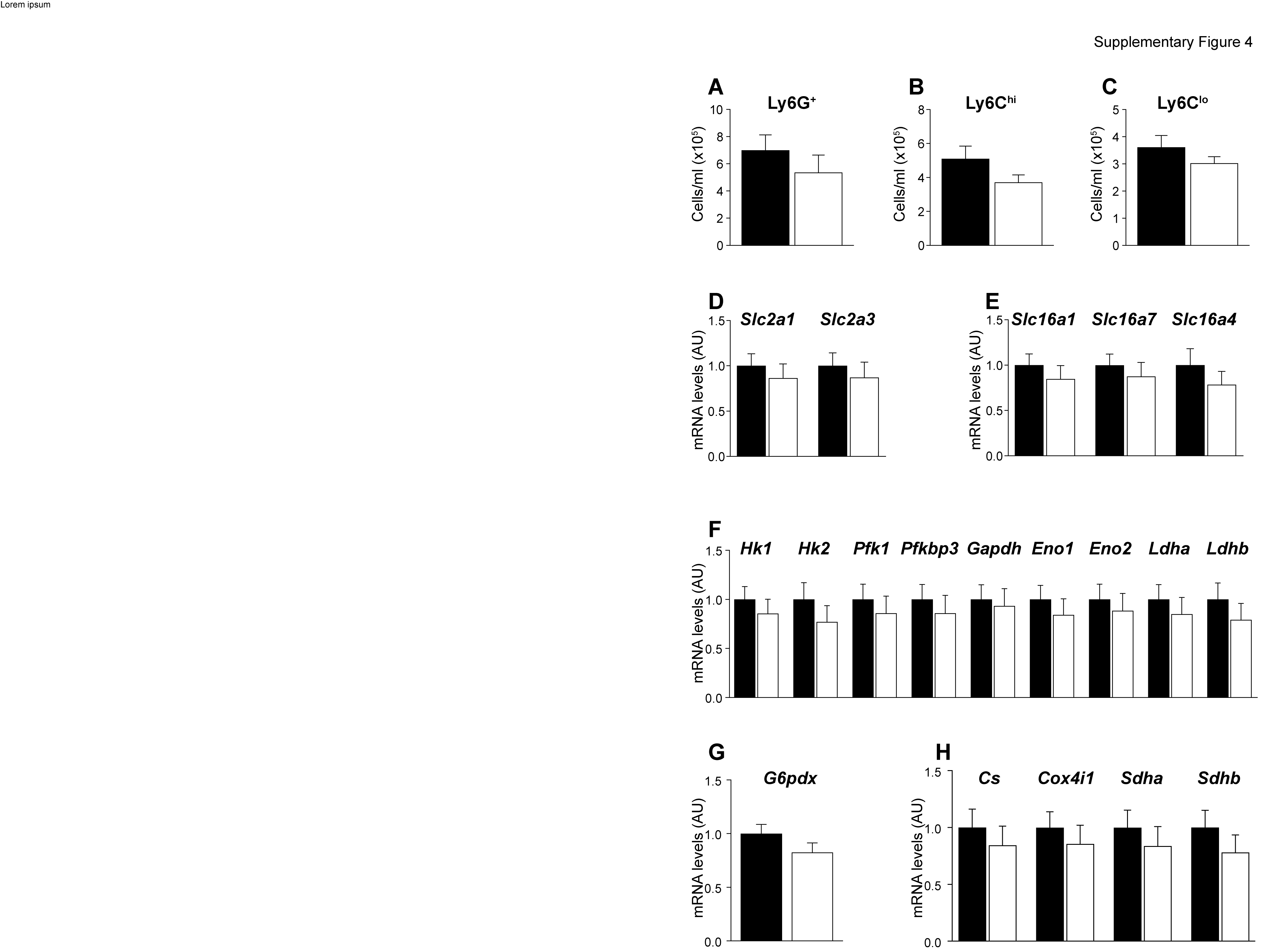
*

***Supplementary Figure 4. Inflammatory and hippocampal metabolic status of naïve Hsd11b1^Del/Del^ and C57BL/6 mice.***

Naive C57BL/6 (black bars) and *Hsd11b1^Del/Del^* mice (white bars) were euthanised by CO_2_ asphyxiation. (A) Numbers of circulating neutrophils (Ly6G^+^ cells) and monocytes (Ly6C^hi^ and Ly6C^lo^) were quantified by flow cytometry (expressed per ml of blood). RT-qPCR was used to quantify hippocampal levels of mRNA encoding (D) glucose transporters (*Slc2a1*/GLUT1, *Slc2a3*/GLUT3), (E) lactate transporters (*Slc16a1*/MCT1, *Slc16a7*/MCT2, *Slc16a4*/MCT4), (F) glycolytic enzymes, (G) glucose 6-phosphate dehydrogenase (*G6pdx*), the rate limiting enzyme of the pentose phosphate pathway and (H) mitochondrial enzymes. Levels of specific mRNAs are expressed relative to the levels of *Hprt* and *Actb* mRNA (used as internal standards), with levels of each mRNA in C57BL/6 mice arbitrarily set to 1. Data are means ± SEM and were analysed by unpaired *t*-test with Welch’s correction (for unequal variance), n=7-8.


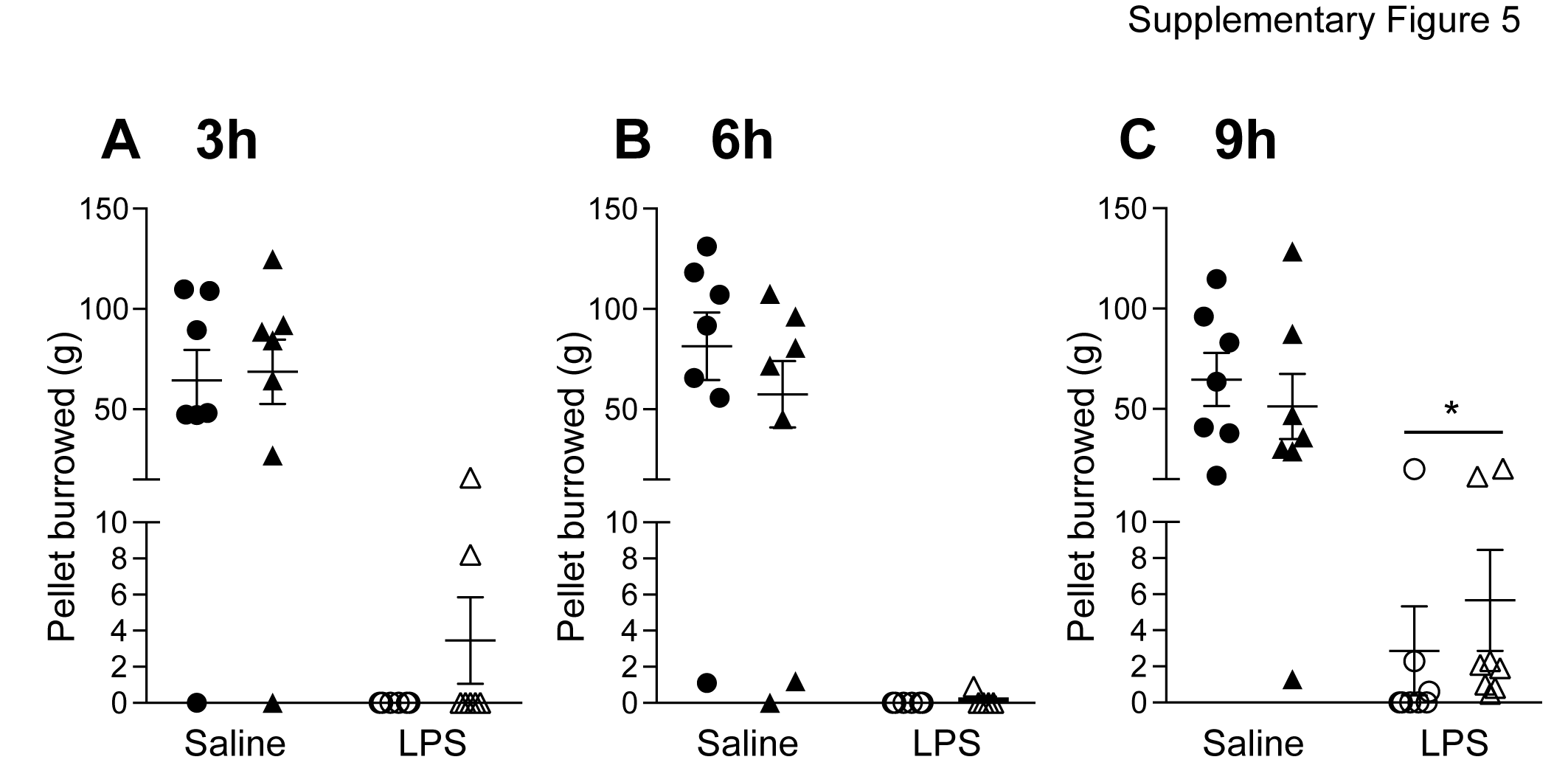


***Supplementary Figure 5. Burrowing activity, a measure of sickness behaviour, is reduced in Hsd11b1^Del/Del^ and C57BL/6 mice following LPS administration.***

C57BL/6 and *Hsd11b1^Del/Del^* mice were injected with vehicle (0.9% saline) or 100µg/kg LPS and euthanised 3h, 6h or 9h later, immediately following a 2h assay of burrowing activity to assess sickness behaviour. Burrowing activity was measured by weight of pellets removed (incremental data) by C57BL/6 (circles) and *Hsd11b1^Del/Del^* mice (triangles): (A) 3h, (B) 6h or (C) 9h after saline (closed symbols) or LPS injection (open symbols) are shown. Symbols show data from individual mice and horizontal bars are medians ± interquartile ranges. Data were first ranked using a Kruskal-Wallis test (for discontinuous data) with pair-wise comparison by Mann-Whitney test; ^*^p<0.05, n=7-8.


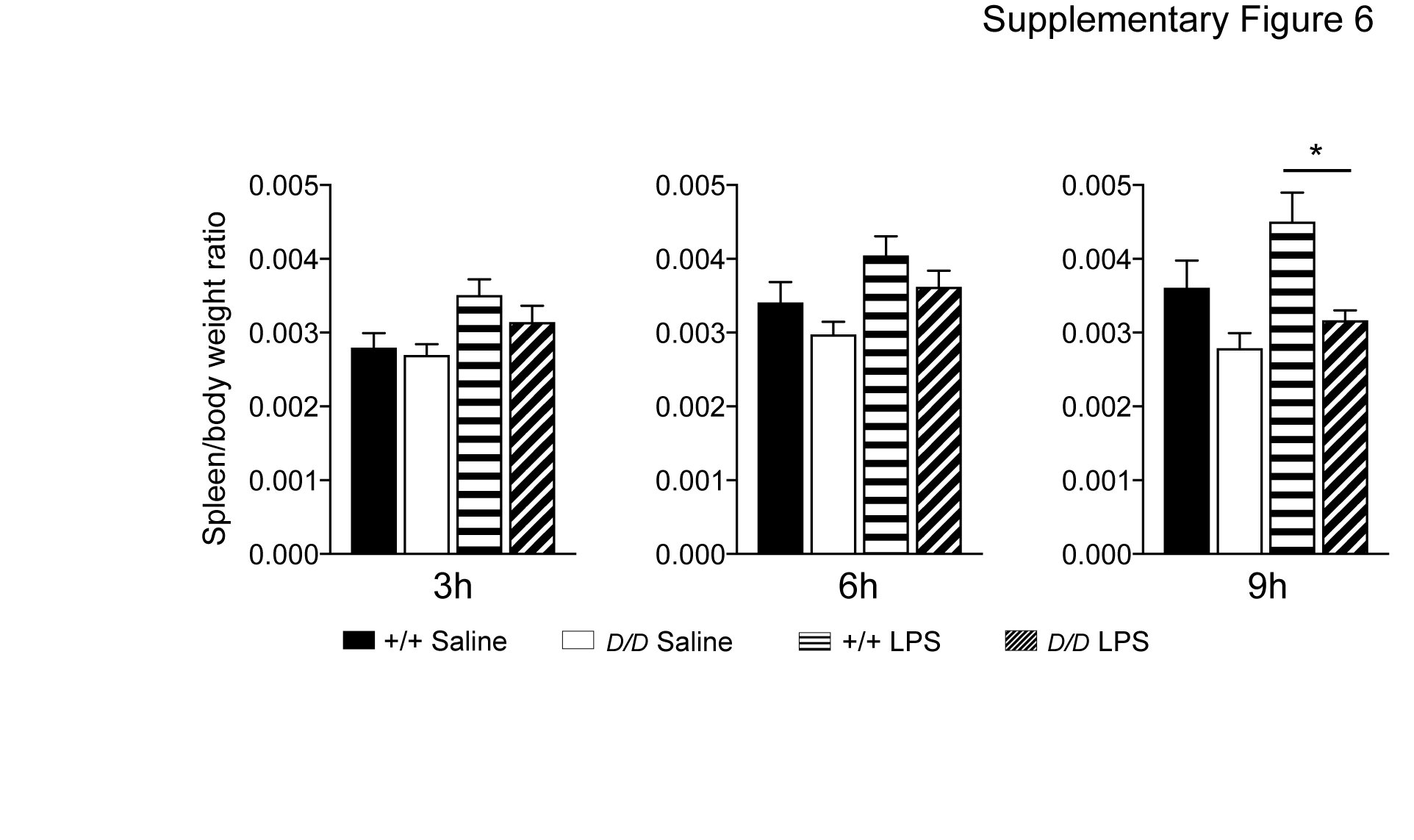


***Supplementary Figure 6. Spleen weights in Hsd11b1^Del/Del^ and C57BL/6 mice following LPS or vehicle administration.***

*Hsd11b1^Del/Del^* and C57BL/6 mice were injected with vehicle (0.9% saline) or 100µg/kg LPS and euthanised 3h, 6h or 9h later. Spleen weight was measured and is expressed relative to body weight. Data are means ± SEM. Two way ANOVA showed a significant effect of treatment at all time points (p<0.05) and a significant effect of genotype at 9h. Post-hoc Tukey’s multiple comparisons test: *p<0.05, n=7-8. Black bars, vehicle-treated C57BL/6 mice (+/+ Saline); white bars, vehicle-treated *Hsd11b1^Del/Del^* mice (*D/D* Saline); horizontal hatched bars, LPS-treated C57BL/6 mice (+/+ LPS); diagonal-hatched bars, LPS-treated *Hsd11b1^Del/Del^* mice (*D/D* LPS).

***
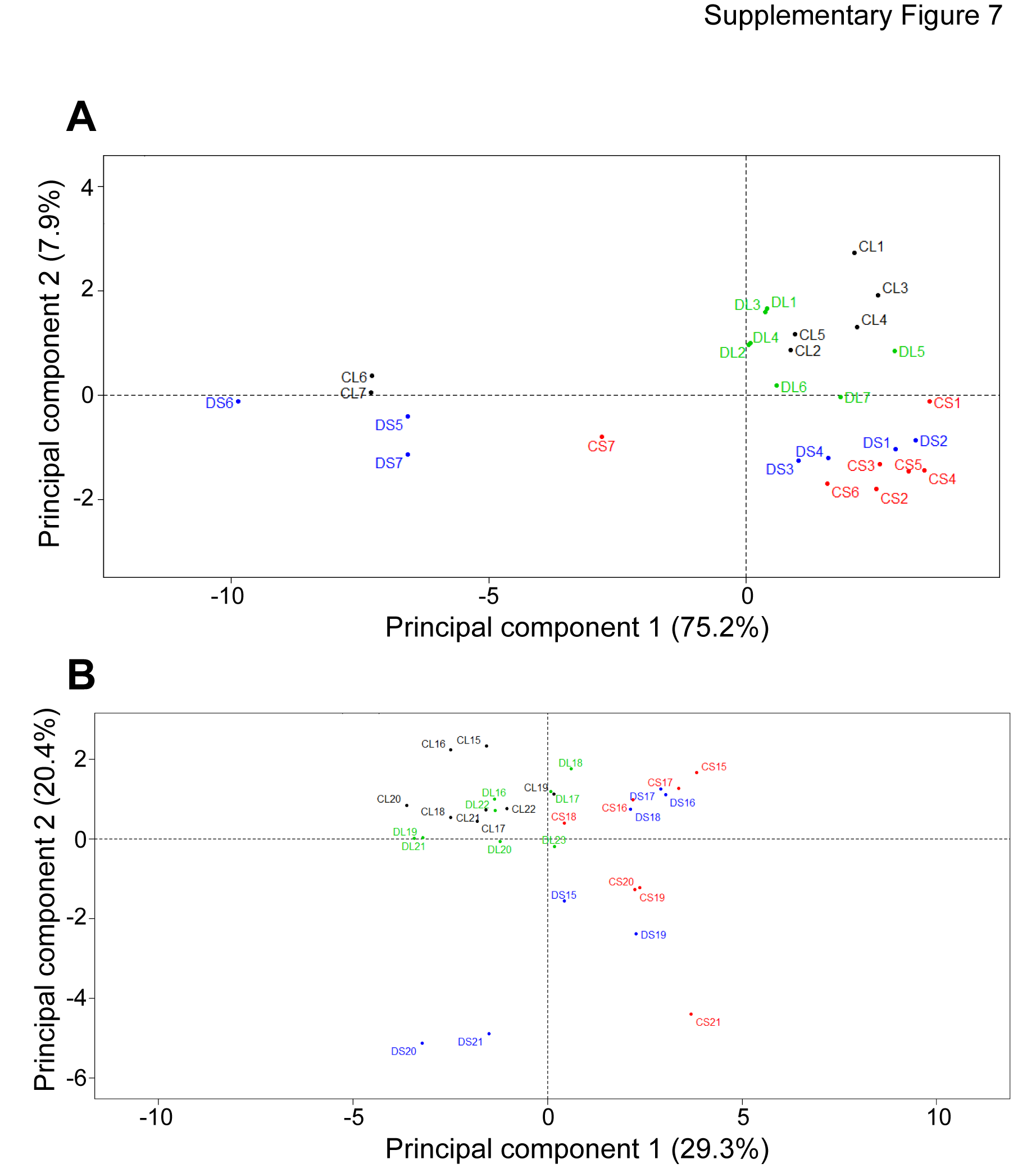
***

***Supplementary Figure 7. Principal component analysis of the levels of mRNA encoding key metabolic transporters and enzymes in Hsd11b1^Del/Del^ and C57BL/6 mice, 3h or 9h following LPS or vehicle administration.***

*Hsd11b1^Del/Del^* and C57BL/6 mice were injected with vehicle (0.9% saline) or 100µg/kg LPS and euthanised 3h or 9h later. Levels of mRNA encoding key metabolic transporters and enzymes in the hippocampus were quantified relative to the mean of *Hprt* and *Actb* mRNA levels by RT-qPCR. Data at 3h (A) and 9h (B) were subject to principal component analysis to investigate differences in expression pattern of specific metabolic genes between the experimental groups. Individual points represent individual mice: CL (black), C57BL/6 + LPS; CS (red), C57BL/6 + saline; DL (green) *Hsd11b1^Del/Del^* + LPS; DS (blue), *Hsd11b1^Del/Del^* + saline.


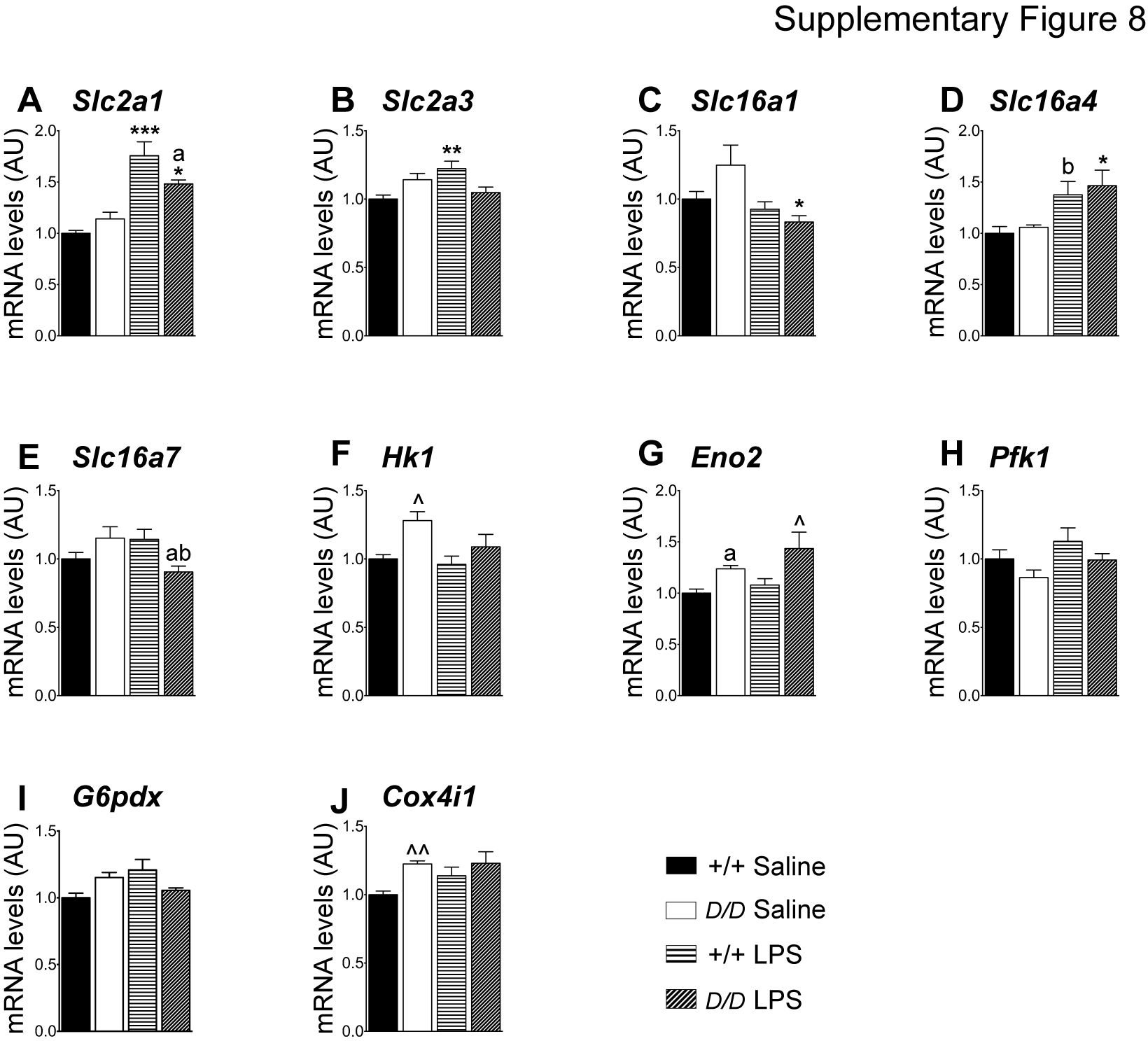


***Supplementary Figure 8. Hippocampal levels of mRNAs encoding genes relevant to energy metabolism in Hsd11b1^Del/Del^ mice, 6h after LPS administration.***

*Hsd11b1^Del/Del^* and C57BL/6 mice were injected with vehicle (0.9% saline) or 100µg/kg LPS and euthanised 6h later. Levels of mRNA encoding transporters: (A) GLUT1 (*Slc2a1*), (B) GLUT3 (*Slc2a3*), (C) MCT1 (*Slc16a1*), (D) MCT4 (*Slc16a4*) and (E) MCT2 (*Slc16a7*); glycolytic enzymes: (F) HK1, (G) Enolase 2 and (H) PFK-1; (I) the pentose phosphate pathway enzyme, glucose-6-phosphate dehydrogenase (*G6pdx*) and (J) subunit 4 of mitochondrial cytochrome c oxidase (*Cox4i1*) were quantified relative to the mean of *Hprt* and *Actb* mRNA levels by RT-qPCR. Data are means ± SEM and are expressed as fold change over levels in vehicle-injected C57BL/6 mice (arbitrarily set to 1). Data were analysed by two way ANOVA, which showed a significant effect of treatment (saline *vs* LPS), p<0.001 (A, D), p<0.05 (C), a significant effect of genotype (C57BL/6 *vs* *Hsd11b1^Del/Del^*), p<0.01 (F, G, J) and a significant interaction; p<0.01 (B, E), p<0.05 (A). Post-hoc tests were Tukey’s (significant effect of treatment: ***p<0.001, **p<0.01, *p<0.05 and of genotype: ^^p<0.01, ^p<0.05) or Fisher’s LSD (significant effect of treatment: ^b^p<0.05 and of genotype: ^a^p<0.05); n=6-7. Black bars, vehicle-treated C57BL/6 mice (+/+ Saline); white bars, vehicle-treated *Hsd11b1^Del/Del^* mice (*D/D* Saline); horizontal hatched bars, LPS-treated C57BL/6 mice (+/+ LPS); diagonal-hatched bars, LPS-treated *Hsd11b1^Del/Del^* mice (*D/D* LPS).

***Supplementary Results and Discussion: Genes relevant to energy metabolism are differentially expressed in the hippocampus of Hsd11b1^Del/Del^ mice, 6h after LPS***

The differential expression of mRNAs encoding enzymes that are key to aerobic glycolysis or which control flux through the TCA cycle is described in the main manuscript. Here, we report and discuss additional findings relevant to substrate transport and utilisation.

Levels of mRNA encoding the glucose transporters GLUT1 (*Slc2a1*) and GLUT3 (*Slc2a3*) were induced by LPS in control mice, whereas only *Slc2a1* was increased in *Hsd11b1^Del1/Del1^* mice (Supplementary Figure 8A, B). This suggests glucose uptake across the blood-brain barrier is increased during inflammation, to a lesser extent in *Hsd11b1^Del1/Del1^* mice than in controls. However, steady-state hexose and hexose-6-phosphate levels were unaffected by LPS treatment (Table 1), suggesting that any additional glucose taken up is rapidly phosphorylated and retained for metabolism. Inter- and intracellular lactate shuttles permit the use of lactate as an oxidative fuel (5, 6). The astrocyte MCT4 (*Slc16a4*) transporter rapidly exports lactate derived from glycogen breakdown, for uptake by neuronal MCT2 (*Slc16a7*). The uptake transporter, MCT1 (*Slc16a1*), is highly expressed in glia and endothelial cells. LPS treatment increased levels of *Slc16a4* mRNA in both genotypes (Supplementary Figure 8D), suggesting increased glial export of lactate via MCT-4. In contrast, levels of *Slc16a1* mRNA (encoding MCT1) were decreased in the hippocampus of *Hsd11b1^Del1/Del1^* but not control mice, 6h after LPS (Supplementary Figure 8C). Consistent with the known correlation between MCT1 expression and extracellular lactate levels (6), the pattern of *Slc16a1* expression across the groups was mirrored in the pattern of lactate concentration in the hippocampus, with lower levels, specifically in *Hsd11b1^Del/Del^* mice, 6h after LPS (Figure 6B).

Glucose entering the cell is rapidly phosphorylated. Although levels of *Hk1* (encoding the predominant hexokinase in brain) and *Eno2* mRNA (encoding neuron-specific enolase) were higher in *Hsd11b1^Del/Del^* mice than controls, neither was affected by LPS (Supplementary Figure 8F, G). The product of PFKFB3, fructose 2,6-bis phosphate, is a potent regulator of PFK1 activity, stimulating glycolysis but levels of *Pfk1* mRNA did not differ between groups (Supplementary Figure 8H). Similarly, there was no difference between groups in expression of *G6pdx* mRNA, encoding glucose-6-phosphate dehydrogenase, the enzyme controlling entry to the pentose phosphate pathway (Supplementary Figure 8I).

*Cox4i1* encodes a subunit of cytochrome c oxidase, the final enzyme in the mitochondrial electron transport chain. Consistent with an increase in mitochondrial oxidative metabolism and reserve respiratory capacity in *Hsd11b1^Del/Del^* mice and similar to *Cs* (Figure 5F), 2-way ANOVA showed a significant effect of genotype on *Cox4i1* mRNA levels, which were increased in *Hsd11b1^Del/Del^*, compared to C57BL/6 mice following vehicle injection (Supplementary Figure 8J).


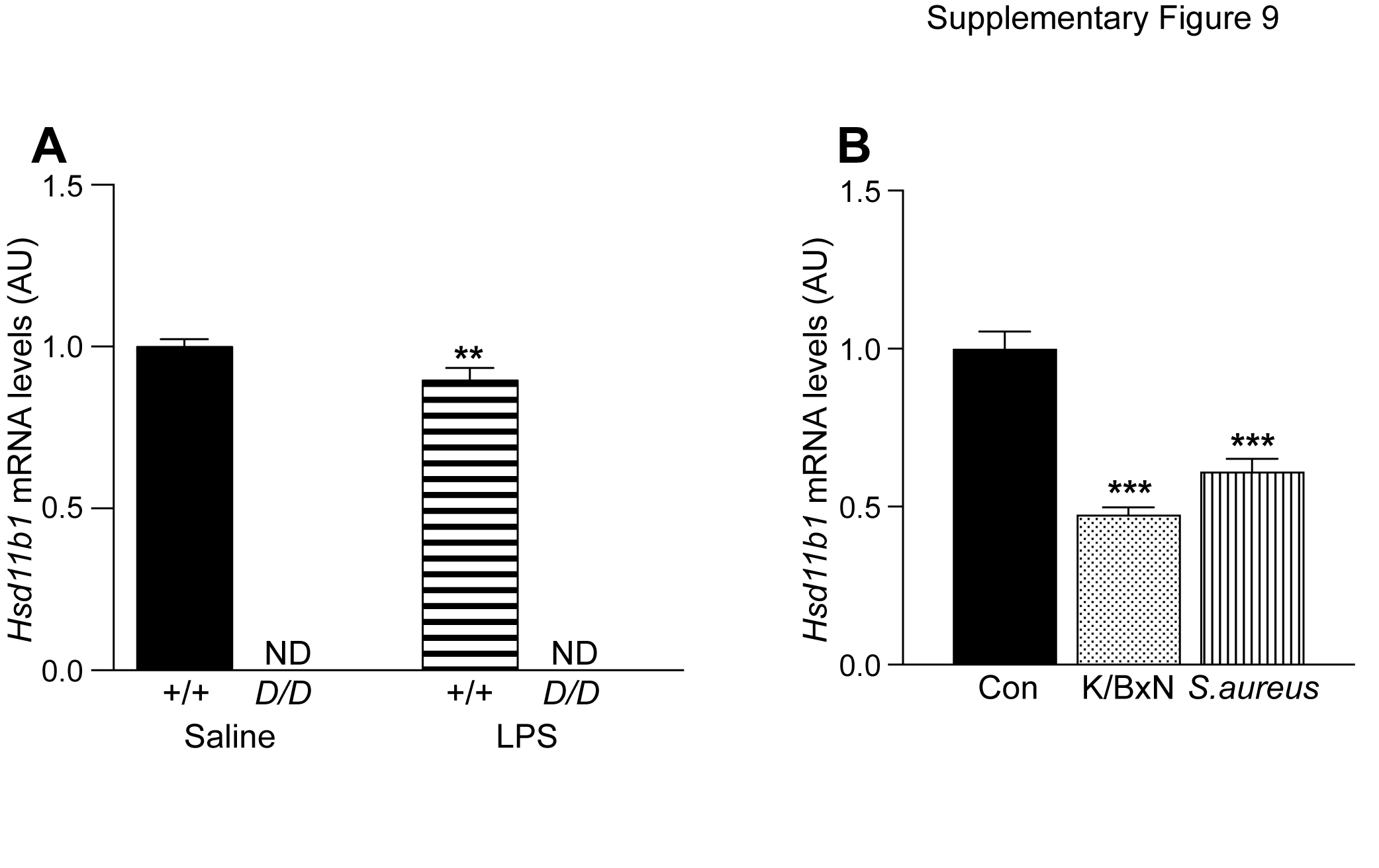
***Supplementary Figure 9. Hsd11b1 mRNA levels are reduced in C57BL/6 mice by acute inflammation.***

(A) C57BL/6 (+/+) and *Hsd11b1^Del/Del^* (*D/D*) mice were injected with vehicle (0.9% saline, black bar) or 100µg/kg LPS (horizontal hatched bar) and euthanised 9h later. Hippocampal *Hsd11b1* mRNA is expressed in arbitrary units (AU) relative to the levels of *Hprt* and *Actb* mRNA (mean, used as internal standard), with levels of *Hsd11b1* mRNA in saline injected C57BL/6 mice arbitrarily set to 1. Data are means ± SEM. Students *t*-test was used to compare *Hsd11b1* mRNA levels in saline and LPS injected C57BL/6 mice: **p<0.01; n=7-8. ND, not detected. (B) Peripheral inflammation was induced in C57BL/6 mice either by installation of *Staphylococcus aureus* into the lungs or i.p. injection of arthritogenic K/BxN serum. Mice were euthanised either 24h (for *S. aureus*) or 15d later (K/BxN serum). Hippocampal *Hsd11b1* mRNA levels are expressed relative to the levels of *Hprt* mRNA (used as internal standard), with levels of *Hsd11b1* mRNA in untreated C57BL/6 control mice arbitrarily set to 1. Data are means ± SEM and were analysed by one way ANOVA followed by Dunnet's multiple comparison test (*vs* untreated C57BL/6 control group), ***p<0.001, n=3-5. Black bar, untreated C57BL/6 control mice; stippled bar, C57BL/6 mice + K/BxN serum; vertical hatched bar, C57BL/6 mice + *S. aureus.*

**REFERENCES**

1 Richardson, R., Batchen, E., Thomson, A., Darroch, R., Pan, X., Rog-Zielinska, E., Wyrzykowska, W., Scullion, K., Al-Dujaili, E.A., Diaz, M. *et al.* (2017) Glucocorticoid receptor alters isovolumetric contraction and restrains cardiac fibrosis. *J Endocrinol*, **232**, 437-450.

2 Kipari, T., Hadoke, P.W., Iqbal, J., Man, T.Y., Miller, E., Coutinho, A.E., Zhang, Z., Sullivan, K.M., Mitic, T., Livingstone, D.E. *et al.* (2013) 11β-hydroxysteroid dehydrogenase type 1 deficiency in bone marrow-derived cells reduces atherosclerosis. *FASEB J*, **27**, 1519-1531.

3 Rog-Zielinska, E.A., Thomson, A., Kenyon, C.J., Brownstein, D.G., Moran, C.M., Szumska, D., Michailidou, Z., Richardson, J., Owen, E., Watt, A. *et al.* (2013) Glucocorticoid receptor is required for fetal heart maturation. *Hum Mol Genet*, **22**, 3269-3282.

4 Zhang, Z., Coutinho, A.E., Man, T.Y., Kipari, T.M.J., Hadoke, P.W., Salter, D.M., Seckl, J.R. and Chapman, K.E. (2017) Macrophage 11β-HSD1 deficiency promotes inflammatory angiogenesis. *J Endocrinol*, **234**, 291-299.

5 Draoui, N. and Feron, O. (2011) Lactate shuttles at a glance: from physiological paradigms to anti-cancer treatments. *Dis Model Mech*, **4**, 727-732.

6 Brooks, G.A. (2009) Cell-cell and intracellular lactate shuttles. *J Physiol*, **587**, 5591-5600.
